# Integrative Analysis of Epigenetic Modulation in Melanoma Cell Response to Decitabine: Clinical Implications

**DOI:** 10.1371/journal.pone.0004563

**Published:** 2009-02-23

**Authors:** Ruth Halaban, Michael Krauthammer, Mattia Pelizzola, Elaine Cheng, Daniela Kovacs, Mario Sznol, Stephan Ariyan, Deepak Narayan, Antonella Bacchiocchi, Annette Molinaro, Yuval Kluger, Min Deng, Nam Tran, Wengeng Zhang, Mauro Picardo, Jan J. Enghild

**Affiliations:** 1 Department of Dermatology, Yale University School of Medicine, New Haven, Connecticut, United States of America; 2 Department of Pathology, Yale University School of Medicine, New Haven, Connecticut, United States of America; 3 Department of Epidemiology and Public Health, Yale University School of Medicine, New Haven, Connecticut, United States of America; 4 Department of Comprehensive Cancer Center Section of Medical Oncology, Yale University School of Medicine, New Haven, Connecticut, United States of America; 5 Department of Surgery, Yale University School of Medicine, New Haven, Connecticut, United States of America; 6 Department of Cell Biology, New York University School of Medicine, New York, New York, United States of America; 7 San Gallicano Dermatological Institute, Rome, Italy; 8 Molecular Biology, University of Aarhus, Aarhus, Denmark; Institut Pasteur Korea, Republic of Korea

## Abstract

Decitabine, an epigenetic modifier that reactivates genes otherwise suppressed by DNA promoter methylation, is effective for some, but not all cancer patients, especially those with solid tumors. It is commonly recognized that to overcome resistance and improve outcome, treatment should be guided by tumor biology, which includes genotype, epigenotype, and gene expression profile. We therefore took an integrative approach to better understand melanoma cell response to clinically relevant dose of decitabine and identify complementary targets for combined therapy. We employed eight different melanoma cell strains, determined their growth, apoptotic and DNA damage responses to increasing doses of decitabine, and chose a low, clinically relevant drug dose to perform whole-genome differential gene expression, bioinformatic analysis, and protein validation studies. The data ruled out the DNA damage response, demonstrated the involvement of p21^Cip1^ in a p53-independent manner, identified the TGFβ pathway genes *CLU* and *TGFBI* as markers of sensitivity to decitabine and revealed an effect on histone modification as part of decitabine-induced gene expression. Mutation analysis and knockdown by siRNA implicated activated β-catenin/MITF, but not BRAF, NRAS or PTEN mutations as a source for resistance. The importance of protein stability predicted from the results was validated by the synergistic effect of Bortezomib, a proteasome inhibitor, in enhancing the growth arrest of decitabine in otherwise resistant melanoma cells. Our integrative analysis show that improved therapy can be achieved by comprehensive analysis of cancer cells, identified biomarkers for patient's selection and monitoring response, as well as targets for improved combination therapy.

## Introduction

There is growing evidence that tumors are highly heterogeneous and that treatment guided by tumor genotype, epigenotype, and gene expression profile may improve outcome. The implementation of this integrative approach is crucial for the treatment of melanomas, a lethal disease known to be composed of different classes, as revealed by multiple approaches [1]–[4]. Melanomas also harbor mutations that promote the malignant phenotype, such as in *BRAF*, *CDKN2A*, *PTEN*, *CTNNB1*, *NRAS*, *PIK3CA* and *KIT* [5], [6], but except for BRAF (∼50%) and common loss of *CDKN2A*, these mutations exist in a minor portion of melanoma specimens (10% or less). Nevertheless, mutation analysis is already a part of clinical operating procedures, such as in selecting patients for treatment with PLX4032 (Plexxikon Inc.) and Imatinib (Gleevec, Novartis Pharmaceuticals), inhibitors specific for activated BRAF and KIT, respectively.

Epigenomic dysregulation, such as methylation of DNA at CpG rich islands in promoter regions and histone-tail modifications are common in cancer cells [7]–[9] as well as in melanomas [10]. Aberrant DNA methylation is the cause for downregulation of tumor suppressors, apoptotic factors, DNA repair enzymes, adhesion molecules and immunomodulators involved in malignant progression of various cancers [9], [11], [12]. These epigenomic marks are cell- and tumor- type specific, they are reversible and thus are targets for cancer therapy [13], [14]. For example, the well-characterized DNA methyltransferase inhibitor decitabine (5-Aza-2′-deoxy-cytidine, Aza), is active as a single agent in myelodysplastic syndrome, acute myeloid leukemia (AML) and chronic myeloid leukemia (CML), and has also been in clinical trials for solid tumors, such as melanomas, but with disappointing results. There are probably multiple factors behind lack of responsiveness, among them the instability of the drug, failure to achieve optimal concentration, or failure to exert the intended activity [15]. However, Aza can sensitize cells to chemotherapeutic [16] and immunotherapeutic drugs [17]–[19], and combination therapy with existing or novel DNA demethylating agents can become more efficient in treating solid tumors.

Consequently, there is a need for a better understanding of the molecular effects of clinically relevant concentrations of decitabine, and to identify markers that predict tumor sensitivity and/or can be used to monitor drug efficacy. In the studies described here we explored the mechanism of action of low-dose decitabine on melanoma cells that are relatively sensitive or resistance to this drug, assessed global gene expression, conducted extensive bioinformatic analysis for biomarker discovery, investigated the contribution of somatic mutations to decitabine resistance, validated some of the changes at the protein level, performed functional analyses, and explored synergistic treatment based on susceptibility of key proteins to proteasomal degradation. We demonstrate that the growth inhibitory effects of low-dose decitabine cannot be attributed to DNA damage response but rather to reconstitution of growth suppressive pathways; activating mutation in β-catenin can confer resistance; and Bortezomib can re-sensitize resistant cells to decitabine.

## Results

### Cell proliferation and apoptotic responses to Aza

**Figure 1:**
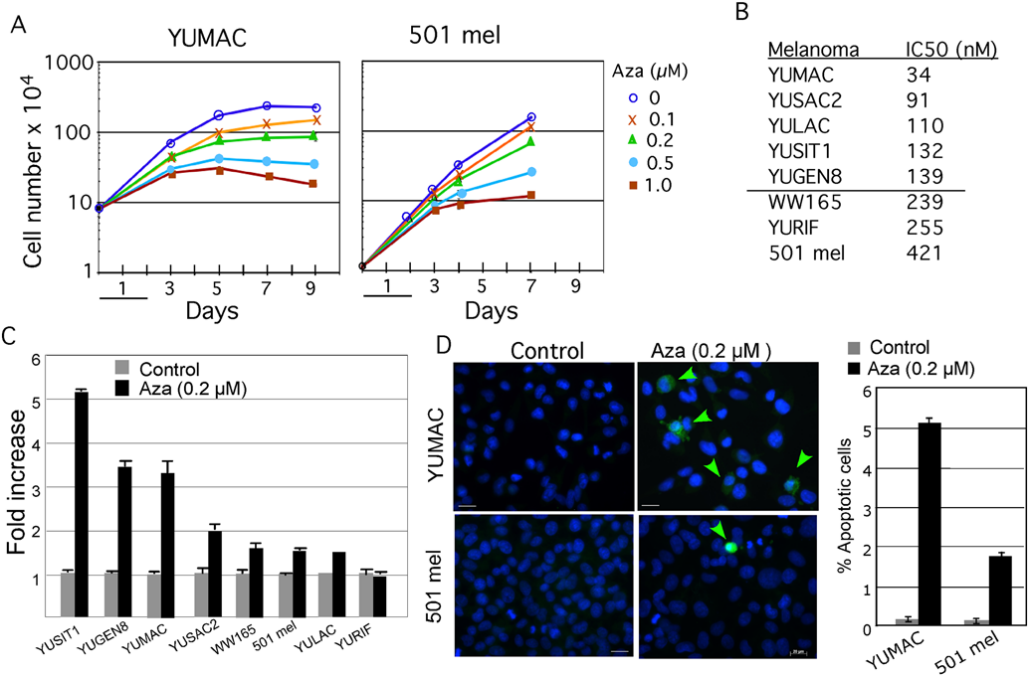
Cellular responses to Aza. Panel A. Growth arrest in response to Aza. Melanoma cells were untreated or treated with increasing concentrations of Aza for 2 days (under line), released into regular growth medium and counted at 2–3 days intervals. The figure shows representative growth curves of a sensitive (YUMAC) and resistant (501 mel) melanoma cell strains of two biological replicates. Supplemental data provide the growth curves (Figure S1) and the population doubling time (Table S1) of all cell strains. Panel B. Aza IC50 response. The vertical line separates the designated sensitive (top) and resistant cell cells (bottom). Panel C. Apoptosis in response to low-dose Aza (0.2 µM) measured by the Caspase-Glo 3/7 assay kit. Panel D. Apoptosis in response Aza (0.2 µM) detected by immunofluorescence with anti-caspase-3 active rabbit antibodies (green arrows point at green fluorescing apoptotic cells). The cell nuclei are stained with DAPI (blue). Bars indicate 20 µm. The histogram shows percent apoptotic cells measured by counting the number of active caspase-3 positive green fluorescing cells in 10 independent microscopic fields representing about 800 cells each. The cell base assay shows a lower percentage of apoptotic cells in response to Aza compared to Panel C because large numbers of affected cells detached during the staining and washing procedures.

Aza dose-response analyses revealed that five of the melanoma cell strains (YUMAC, YUSAC2, YULAC, YUSIT1, and YUGEN8) were relatively sensitive to the drug with IC50 ranging between 13–135 nM, and the other three (WW165, YURIF and 501 mel) were relatively resistant with IC50 ranging between 233–417 nM as determined by cell proliferation assays (**Figure 1A, B** **, Figure S1 and Table S1**). The differences between drug responses were not due to the number of passages in cultures (YUSIT1, YUSAC2, WW165 and 501 mel cells were long-term cultures whereas YUMAC, YULAC and YURIF were melanoma cells freshly cultured from different tumors). Nor were BRAF, N-Ras activation or PTEN mutation/loss responsible for the differences, because all cell strains expressed the activated BRAF kinase, none harbored N-Ras mutation, and PTEN expression was lost in only two cell strains, while one PTEN-positive cell strain carry a known variant (Pro38Ser) (**Table 1**). The stage of the original melanoma tumor was also not a factor because the resistant WW165 melanoma cells were established from primary melanoma, and all the others from metastatic lesions.

**Table 1:** Sources of patient's derived melanoma cells.

Melanoma	Gender/age	Stage/site	BRAF status	PTEN
WW165	F/62	Primary melanoma, 2.25 mm	V600K (GTG->AAG)	WT, Present*
YUMAC	M/68	IV, Soft tissue metastasis, right thigh	V600K (GTG->AAG)	WT, Null (no protein)
YUGEN8	F/44	IV, Brain metastasis	V600E (GTG->GAG)	Null (no gene transcripts)
YUSAC2	M/57	IV, Soft tissue metastasis, left neck	V600E (GTG->GAG)	WT/LOH (Present)
YUSIT1	M/67	Metastatic melanoma	V600K (GTG->AAG)	WT (Present)
YULAC	F/66	IV, Soft tissue metastasis, neck	V600K (GTG->AAG)	P38S/LOH (C1143T)
YURIF	M/53	IV, Soft tissue metastasis, right thigh	V600K (GTG->AAG)	LOH Present
501 mel	Not known	Lymph node metastasis	V600E (GTG->GAG)	WT, Present

* Present indicates normal levels of gene transcripts and protein expression compared to normal melanocytes. There was no induction of PTEN mRNA after Aza treatment.

Apoptosis in response to Aza treatment was also variable and generally in agreement with the proliferative responses (**Figure 1C**). Low doses of decitabine (0.2 µM) elicited an intense apoptotic response in YUSIT1, YUGEN8 and YUMAC (3–5 fold increase over control), intermediate response in YUSAC2, WW165 and 501 mel (1.5–2-fold increase over control), and no response in YURIF melanoma cells. Immunostaining with activated caspase-3 antibodies showed that the differences between resistant and sensitive cells were at the level of the number of cells undergoing apoptosis (**Figure 1D**).

We chose 2-day treatment with low-dose Aza (0.2 µM) followed by one-day recovery in fresh growth medium for all subsequent experiments, because this concentration of decitabine discriminated well between sensitive and resistant cells based on cell proliferation assays (**Figure 1A** **and Figure S1**) and is also the one likely to be reaching solid tumors *in vivo* in patients treated with this agent [15]. Decitabine has very short circulating half-life, and patients receiving 30–40 mg/m^2^ per 24 hours (twice the current approved dose) by continuous intravenous infusion for 72 hours achieved plasma concentrations of 0.12 to 0.16 µM.

### The DNA damage response cannot account for low-dose Aza induced growth arrest

We first assessed whether the DNA damage response is the basis for growth arrest in response to low-dose Aza treatment in our panel of melanoma cell strains, because Aza at even 0.1 µM can induce DNA damage in human lung cancer cell lines [20]–[23], and concentrations of ∼1 µM and above also activate p53, resulting in p21^Cip1^ induction and cell cycle arrest [20]–[22]. We performed the Comet assay which measures DNA damage at the level of individual cells. This test revealed that 0.5 µM and 1.0 µM, but not 0.2 µM Aza induced DNA damage in the Aza sensitive YUMAC, but not the resistant YURIF melanoma cells (**Table 2**). Furthermore, additional tests excluded the induction of double-strand break DNA repair and activation of cell-cycle checkpoints after low-dose Aza, because: a) there were no changes in the levels of phosphorylation of proteins known to transmit the ATR (ataxia telangiectasia mutated (ATM) and ATM and Rad-3 related) response, CHK1, and gamma-H2AX, reported to be activated in response to high dose Aza (1–10 µm) [20]–[22] (data not shown); b) there was no accumulation of p53 phosphorylated forms (Ser37 and Ser20); and c) there was no induction of BAX, that was expressed at equal levels in these cells, or additional p53 signature genes, such as GADD45.

**Table 2:** DNA damage in response to Aza as measured with the comet assay YUMAC.

Melanoma	Aza (µM)	%DNA in Tail±SE	%Tail Length±SE
YUMAC	0	2.36±0.25	11.190±0.66
	0.2	1.13±0.12	8.55±0.56
	0.5	5.20±0.41	18.67±0.73
	1.0	4.35±0.24	16.79±0.50
	100 µM H_2_O_2_	32.89±1.30	43.47±0.75
YURIF	0	11.73±0.44	34.44±0.77
	0.2	11.37±0.49	34.35±0.87
	0.5	6.94±0.48	27.03±0.93
	1.0	8.27±0.49	28.32±0.80
	100 µM H_2_O_2_	69.99±1.49	72.48±0.95
YUSAC2	0	6.30±0.32	23.52±0.62
	0.2	7.03±0.49	24.26±0.77
501 mel	0	9.24±0.39	26.68±0.58
	0.2	6.43±0.37	22.66±0.65
WW165	0	11.58±0.41	29.62±0.60
	0.2	5.80±0.38	19.50±0.70
YUGEN8	0	8.22±0.45	25.99±0.73
	0.2	6.19±0.50	23.73±0.80
YULAC	0	8.48±0.38	25.46±0.60
	0.2	4.85±0.25	20.49±0.57
YUSIT1	0	7.38±0.33	23.76±0.69
	0.2	7.83±0.39	24.63±0.62

Melanoma cells were untreated or treated with Aza for 2 days, harvested after one-day recovery in standard growth medium, and subjected to the Comet assay. Cells were examined with fluorescence microscope, photographed, and analyzed with CASP software (http://casp.sourceforge.net). The percentage of the DNA tail area was divided to total DNA area for each cell, and percentage of DNA tail length divided to total DNA length was counted. The data represent averages from 100–120 cells±SE; YURIF DNA damage response is a representative of two biological replicates with similar results. Treatment with hydrogen peroxide (100 µM) for 10 minutes on ice was used as positive control.

We concluded that 0.2 µM did not cause DNA damage. We therefore, explored gene reactivations that lead to changes in specific signaling pathways as the mechanism of Aza cellular responsiveness, and attempted to identify markers by interrogating specific genes revealed by the bioinformatic analyses.

### Whole-genome gene expression profiling in response to Aza

**Figure 2:**
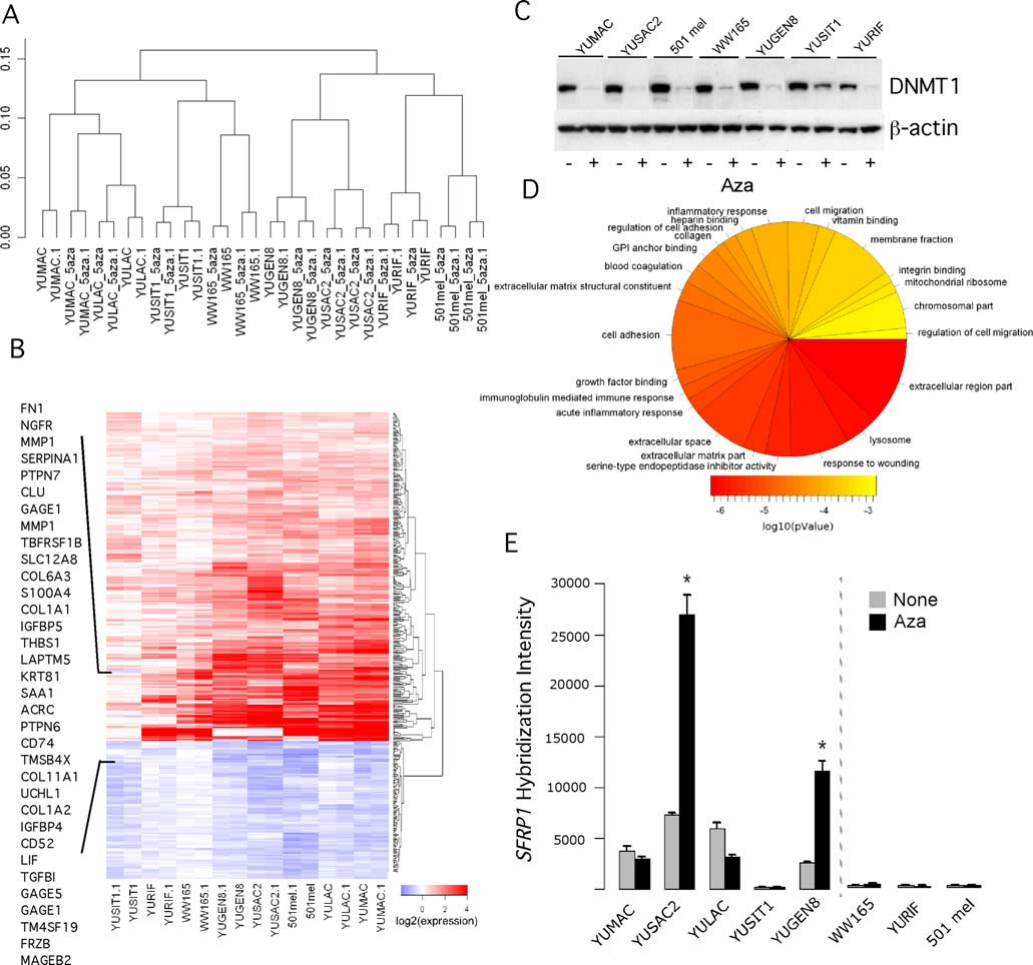
Bioinformatic analysis of whole genome expression arrays. Panel A. Unsupervised hierarchical clustering of absolute intensity values. The vertical scale indicates 1-pearson's correlation coefficients as a measure of similarity. Panel B. Heatmap of differentially expressed sequences after treatment with low-dose Aza. Panel C. DNMT1 expression at the end of 3-days treatment with Aza (0.2 µM). Cell extracts were subjected to Western blot with anti-DNMT1 antibodies. The same membrane was successively blotted with anti-b-actin antibodies as a measure for protein load in each well. Panel D. Pie chart of the most over-represented Gene Ontology terms (p-value<1e-3); the size is relative to the number of represented genes, and the color represents the enrichment p-value. Panel E. *SFRP1* transcripts in melanoma cell strains as assessed by the oligonucleotide array hybridization. The data represent one of two sequence IDs with similar results. The error bars represent the Standard Deviations (SD). One, two, three stars refer to p-value less than 0.05, 0.01 or 0.001, respectively. We determined p-values by unpaired t-test (Aza vs. Untreated). The broken line in this and all subsequent figures separates sensitive (left hand side) from resistant (right hand side) cell strains.

Unsupervised hierarchical clustering based on similarity of genome-wide expression profiles of the eight melanoma cell strains confirmed low variability between replicate experiments, indicating high quality of results (**Figure 2A**). The clustering of an Aza treated cell strain with its untreated counterpart shows that relatively few genes were affected by Aza treatment. The dendogram also suggested that pre-treatment gene expression by itself harbors important information with respect to Aza responsiveness because sensitive YUMAC, YULAC and YUSIT1, clustered separately from the Aza resistant YURIF and 501 mel melanoma cell strains

We identified 396 sequence ids, representing 292 genes that were differentially expressed across the cell strains following treatment with low-dose Aza (**Figure 2B** **; all microarray data will be deposited in GEO**). At least 50 genes in our list are already known to be regulated by DNA methylation, such as those encoding cancer antigens (a set of *MAGE* and *GAGE*), *H19*, *S100A4*, *IGFBP4*, *UCHL1*, *COL1A2*, *CLU*, *FN1*, and *TGFBI* (**Figure 2B**). We did not see consistent re-expression of genes that have been previously reported to be under epigenetic control and/or reactivated by low concentration of decitabine (0.1 µM) in established uveal melanoma cell lines, such as S100A2 [24], and two melanoma cell lines, such as Apo2L/TRAIL and XAF1 [18], or in response to higher concentrations of decitabine, such as *PTEN*, *HOXB13*, *APC*, *RASSF1A*, *RARB* and *MGMT* [10], [25], [26]. Surprisingly, the Aza sensitive YUSIT1 displayed the lowest intensity of absolute change in gene expression compared to the other melanoma cell strains (**Figure 2B**). This difference might be attributed to higher levels of DNMT1 post Aza treatment in these cells compared to the other cell strains (**Figure 2C**).

The sequence ids have been evaluated for over-representation of functional families based on analysis of enrichment of GeneOntology (GO) terms. Among the highest scoring GO terms were genes associated with the extracellular region, matrix, response to wounding and external stimulus, protease inhibitor activity, and genes associated with acute inflammatory and immune responses (**Figure 2D**). However, we could not find any particular category that could segregate the relatively Aza sensitive from the resistant cell strains.

To better understand cellular responsiveness to the drug, we compared the pre-treatment gene expression levels between sensitive and resistant melanoma cell strains. This analysis uncovered 94 genes (141 sequences) with differential pre-treatment expression. Among them was SFRP1 (Secreted frizzled-related protein 1), the negative regulator of Wnt signaling, whose basal transcript levels were particularly low in the resistant compared to the sensitive melanoma cell strains (**Figure 2E**). Because SFRPs are known to be regulated by DNA methylation and to be reactivated by Aza [27], [28], and Wnt signaling plays an important role in melanoma biology [29], we further explored the role of this pathway in Aza differential responsiveness.

### Activated Wnt/β-catenin/MITF pathway confers resistance to Aza

**Figure 3:**
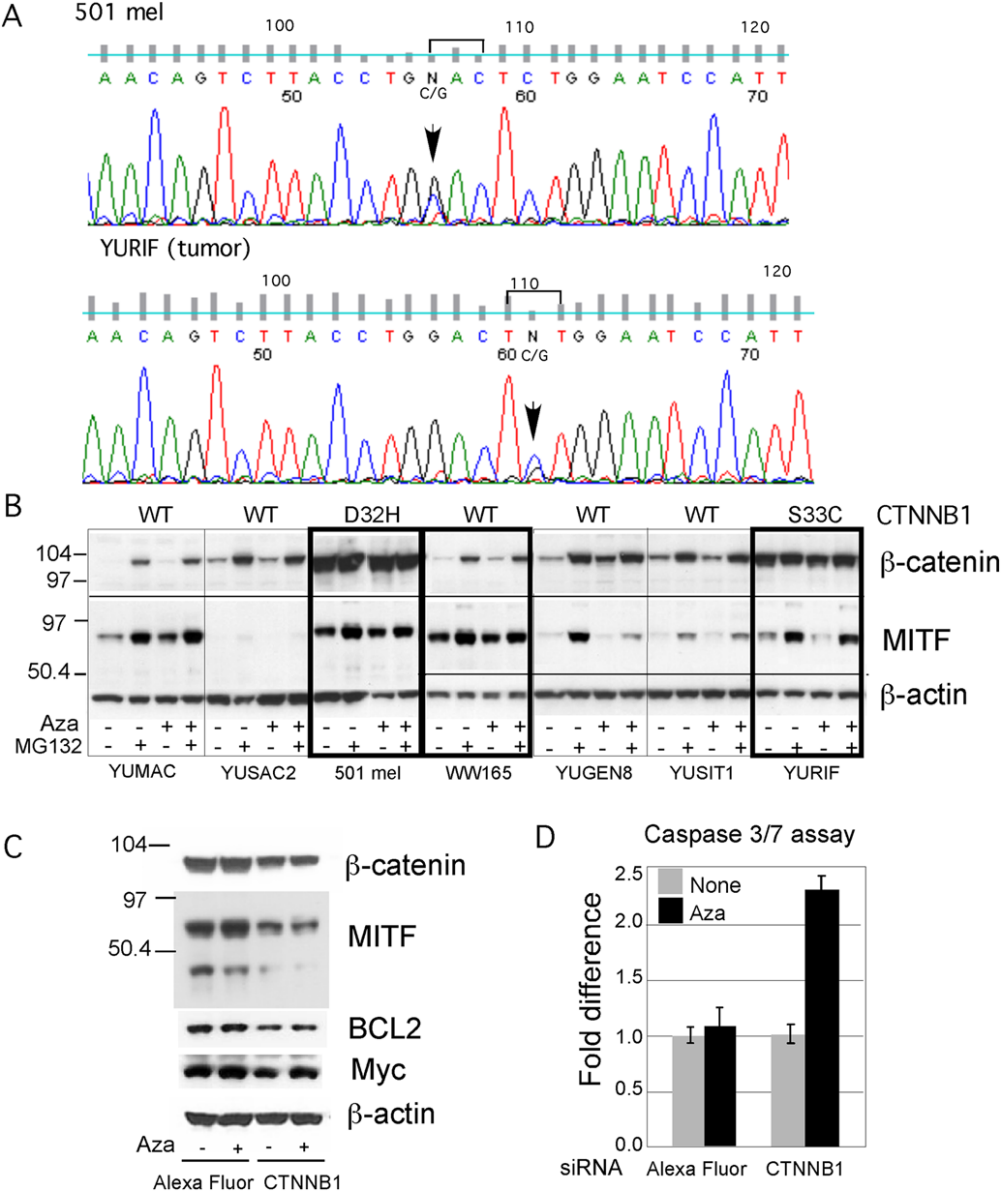
Activated Wnt/β-catenin/MITF pathway confers resistance to Aza. Panel A. Chromatograms showing *CTNNB1* activating mutations in 501 mel cells (GAC/CAC) and YURIF tumor (TCT/TGT) (marked by brackets and arrows), which lead to D32H and S33C mutations, respectively. The same results were obtained with YURIF short term cultured cells. Panel B. Expression of β-catenin and MITF in melanoma cell strains relative to β-actin. *CTNNB1* mutation status for each cell strain is indicated at the top. Panel C. siRNA knockdown of β-catenin and downstream targets. Parallel cultures were untreated or treated with Aza (0.2 µM) for 2 days followed by transient transfection with three different *CTNNB1* siRNA or with Alexa Fluor as a control for one day. Cell extracts were subjected to successive Western blotting with β-catenin, MITF, BCL2, Myc, and β-actin. Panel D. The same cultures as in panel C were assessed for apoptosis employing the Caspase 3/7 assay. Bars indicate SD of 3 replicate wells.

The known role of Wnt downstream target β-catenin compelled us to assess the involvement of β-catenin activating mutation in Aza growth-arrest resistance. Re-sequencing of *CTNNB1* exon 3 revealed D32H, and not S37F substitution reported before [30], in our 501 mel cells, and S33C mutation in YURIF melanoma tumor and cultured cells, whereas the gene in all the other cell strains was normal (**Figure 3A**). YURIF and 501 mel melanoma cells also expressed high constitutive levels of β-catenin (**Figure 3B, β-catenin**), as expected from the increased resistance to proteasomal degradation conferred by the activating mutations. In all the others, β-catenin levels were low but re-appeared in the presence of the proteasomal inhibitor MG132. Treatment with Aza upregulated β-catenin in YUGEN8, and to a lesser extent in YUMAC melanoma cells, in agreement with the arrays hybridization intensities.

Among the β-catenin target genes is *MITF*, encoding a melanocyte-specific transcription factor that can interact with β-catenin to modulate the Wnt signaling and cell growth [29]. *MITF* transcripts were similar or slightly lower than those in normal melanocytes (data not shown), suggesting that the gene was not amplified. However, basal levels of MITF proteins varied among the cell strains, being particularly high in 501 mel and WW165 cells (**Figure 3B, MITF**).

The role of β-catenin in conferring resistance to Aza was further explored by knockdown experiments. Transient CTNNB1-directed siRNA knockdown caused about 70% reduction in β-catenin levels compared to Alexa fluor treated cells (**Figure 3C**) or control siRNA (data not shown). In addition, there was repression of MITF, as well as the anti-apoptotic BCL2. Myc, the other known β-catenin target gene, was downregulated by CTNNB1 knockdown but only in control cells and not in those treated with Aza (**Figure 3C**). Furthermore, downregulation of β-catenin sensitized the 501 mel resistant cells to Aza mediated apoptosis (**Figure 3D**), suggesting that β-catenin signaling shields these resistant melanoma cells from undergoing apoptosis. The results are in agreement with the observations that the *MITF* promoter is responsive to Wnt signaling in melanocytes, that β-catenin binds and trans-activates *MITF*, and that β-catenin induced melanoma growth requires MITF [31]. Unfortunately, similar experiments could not be conducted with YURIF melanoma cells because transfection with CTNNB1-directed siRNA failed to produce any reduction in β-catenin protein (data not shown).

We went on to explore the role of individual reactivated genes and their protein products in Aza responsiveness, focusing on pathways known to induce growth arrest and/or apoptosis, and further examination of known Aza effects other then DNA demethylation.

### Activation of p21^Cip1^ in a p53 independent manner

**Figure 4:**
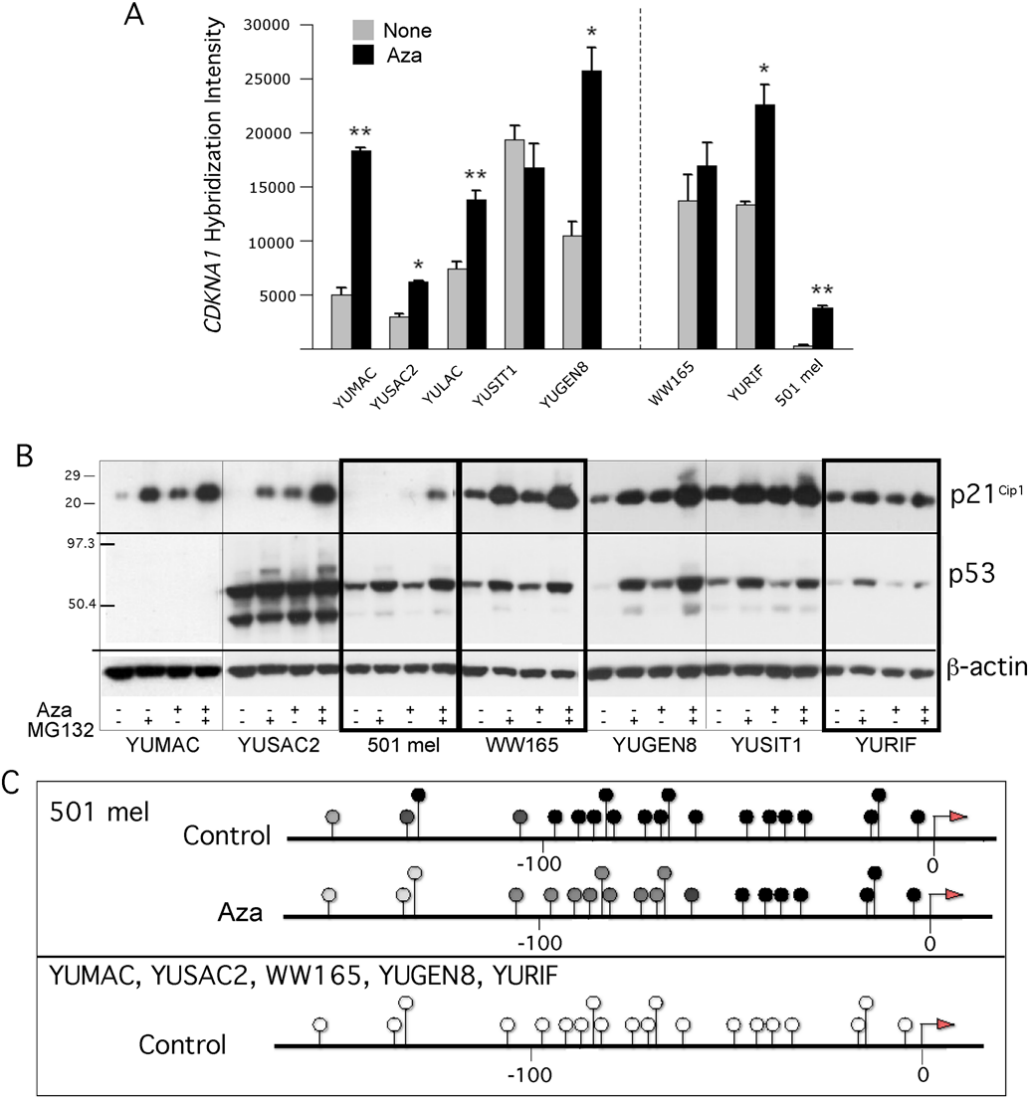
TP53-independet *CDKN1A* reactivation and promoter methylation. Panel A. Reactivation of CDKN1A in melanoma cells in response to Aza (0.2 µM) as revealed by oligonucleotide array hybridization. The data represent one of two sequence IDs with similar results. All other details as in Figure 2 Panel E. Panel B. Expression of p21^Cip1^ and p53, with β-actin serving as a control. Parallel cultures of melanoma cells were untreated (−) or treated (+) with Aza (0.2 µM). MG132 (20 µM) was added 6 h prior to harvesting the cells where indicated (+). The levels of p53 protein were in agreement with gene transcript levels showing that TP53 was inactivated in YUMAC (absolute hybridization intensities values of ∼220, compared to 8,000–12,000 in the melanoma cell strains). Here and in all other Western blots numbers on the left mark the location of prestained protein markers in KDa, heavy and light frames designate Aza resistant and sensitive cells, respectively. Panel C. BS sequencing results of *CDKN1A* proximal promoter (−214 to +20 relative to TSS). Melanoma cells were untreated (control), and Aza (0.2 µM) treated as described in Panel B. Symbols: Arrows indicate the TSS; open and black circles, unmethylated and methylated CG pairs, respectively; dark and light grey circles indicate about 50% and 10% methylated CG, respectively. Numbers on the bottom indicate bp location relative to TSS.

Although the DNA damage response was ruled out, close examination of the oligonucleotide gene expression data showed two-fold increases in *CDKNA1* (encoding p21^Cip1^) transcripts in some melanoma cell strains (**Figure 4A**). Therefore, we assessed p21^Cip1^ levels in cells treated with low-dose Aza, in the absence and presence of MG132, supplemented to the medium 6 hr before harvest in order to prevent proteasomal degradation known to affect the stability of this protein in melanoma cells [32]. Western blotting revealed strong induction of p21^Cip1^ in response to Aza in YUMAC, YUSAC, and YUGEN8 melanoma cells, and less so or not at all in the other cell types. The gene was induced in YUMAC cells null for TP53 (**Figure 4 panels A and B** **, compare p53 to p21^Cip1^**), conforming our previous conclusion that p53 does not mediate growth arrest. All the p53 expressing melanoma cell strains possessed the P72R variant but none carried an inactivating mutation in exon 4 of p53. P72R is a common allele in Caucasians, from which these melanoma tumors were isolated. Therefore, the different levels of p53 in this panel of melanoma cells could not be explained by TP53 genetic variation.

TP53-independent induction of p21^Cip1^ in leukemic cells was attributed to decitabine-induced re-expression of the tumor suppressor p73, an upstream regulator of p21^Cip1^ [33], [34]. However, p73 is not expressed or induced in melanoma cells by Aza. The suppression of *CDKN1A* by methylation of the proximal promoter in senescing fibroblasts [35], prompted us to explore direct methylation and demethylation as the cause for p21^Cip1^ silencing and reactivation, respectively. Indeed, the *CDKN1A* promoter was highly methylated only in 501 mel non-expressing cells, and underwent partial demethylation after treatment with low-dose Aza (**Figure 4C, 501 mel, compare control to Aza**). Interestingly, a cluster of seven CpG dinucleotides proximal to the transcription start site (TSS) remained fully methylated after treatment, which may explain the weak reactivation of p21^Cip1^ in 501 mel cells (**Figure 4A, 501 mel**). In contrast, this promoter region was not methylated in any of the other melanoma cell strains (**Figure 4C**), in agreement with basal p21^Cip1^ gene transcripts and protein (**Figure 4A and B**).

Because low-dose Aza induced *CDKN1A* in several melanoma cell strains in which the promoter was unmethylated, we attempted to identify additional genes that behave in a similar fashion whose reactivation can lead to growth arrest and further explored the mechanism of Aza activity.

### Activation of *CLU* by DNA demethylation

**Figure 5:**
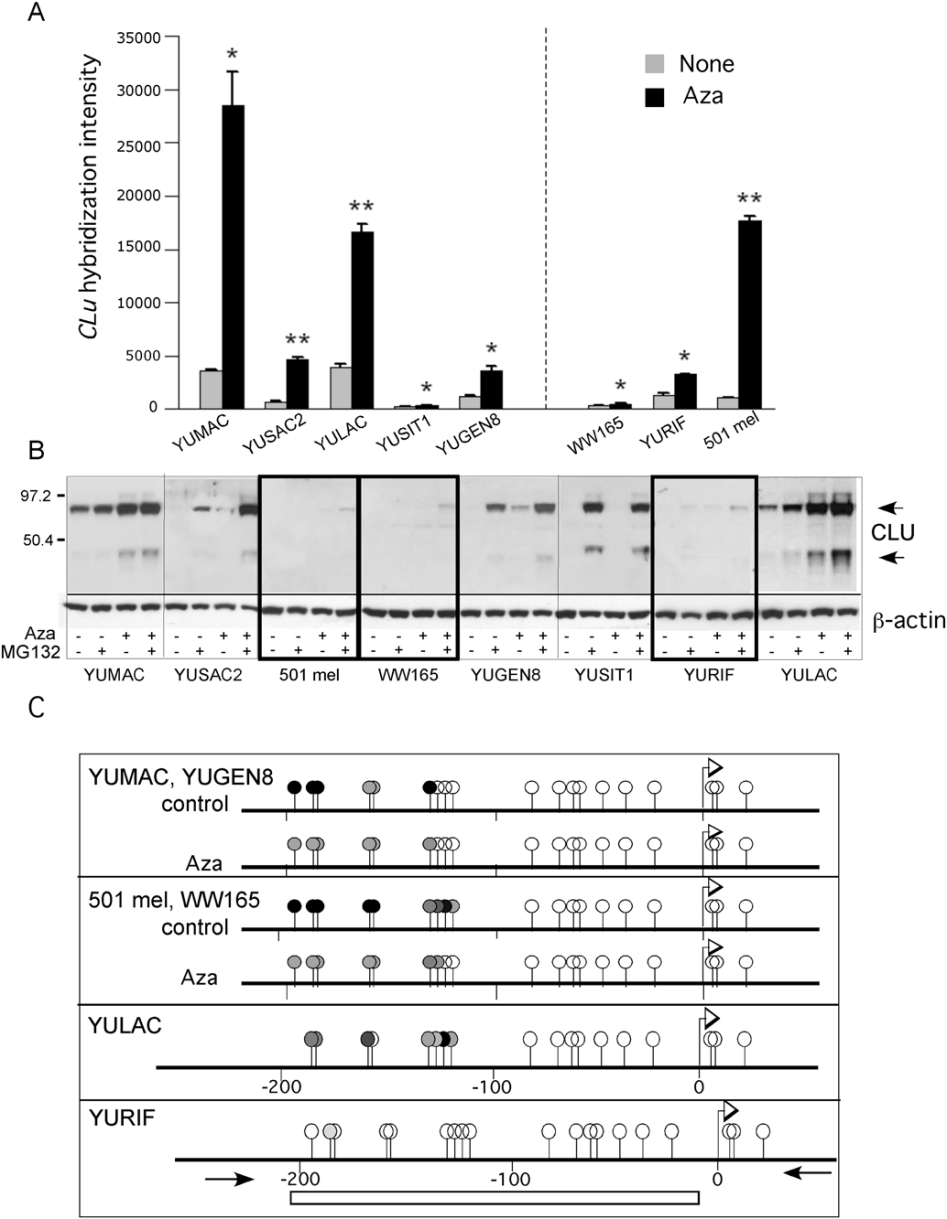
*CLU* reactivation and promoter methylation. Panel A. *CLU* re-expression in melanoma cells in response to Aza (0.2 µM) as assessed by the oligonucleotide array hybridization. The data represent one of three sequence IDs with similar results. All other details as in Figure 2 Panel E. Panel B. Clusterin expression as revealed by Western blots with anti-CLU antibodies. The results are representative of two biological replicas. Panel C. BS DNA sequencing results of the proximal *CLU* promoter and part of first exon regions. The bar indicated the CG island and the arrows the site of primers used for amplification. All other details as in Figure 4.

**Figure 6:**
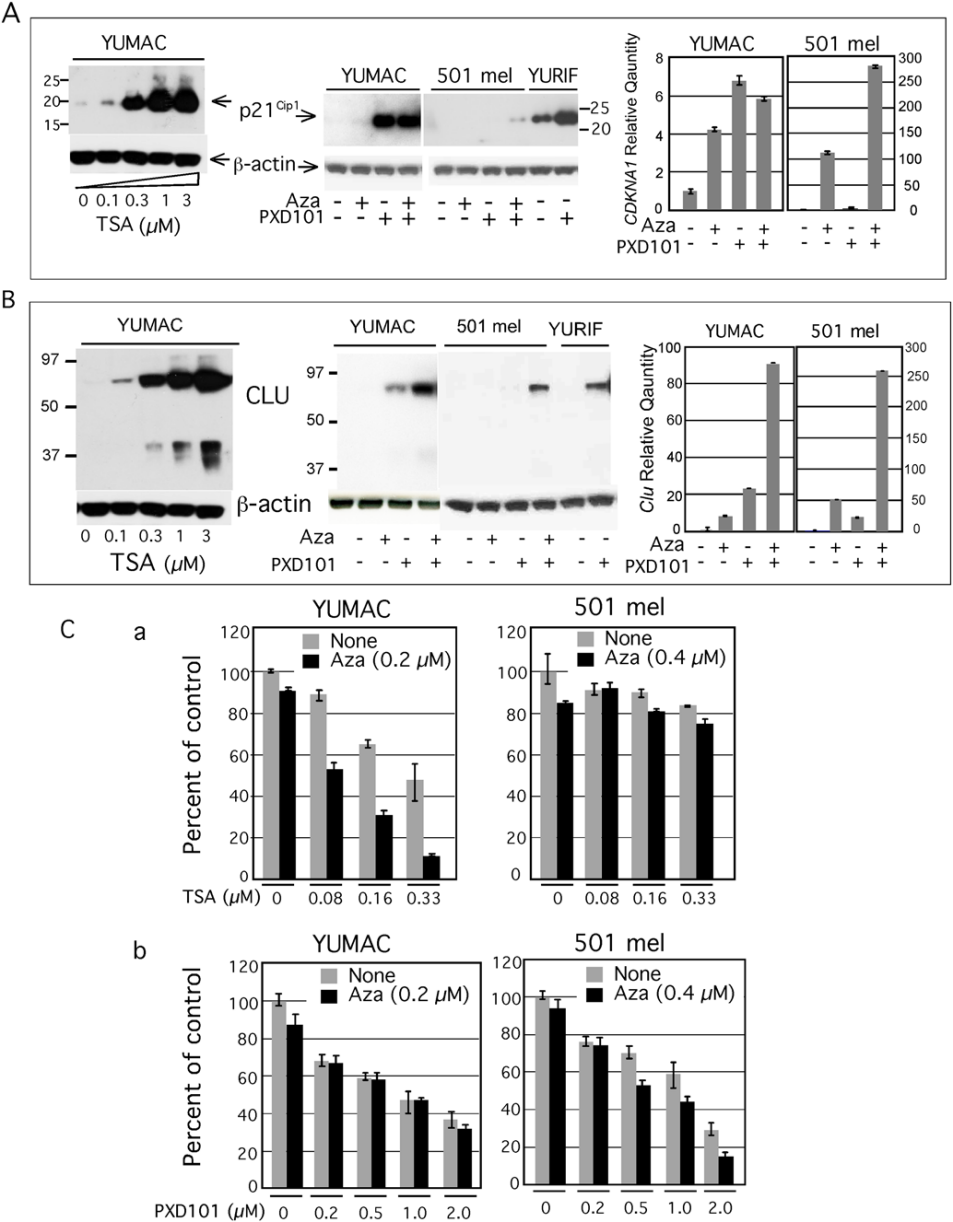
Reactivation of *CDKNA1* and *CLU* by histone acetylation. The Western blots show p21^Cip1^ (Panel A) and CLU (Panel B) expression in YUMAC melanoma cells treated with increasing concentrations of Trichostatin A (TSA) overnight, as revealed by probing with the respective antibodies using β-actin as a control (left panel). Middle panels show expression of p21^Cip1^ and CLU after 2-days treatment with Aza (0.2 µM), where indicated (+) followed by 1-day incubation with PXD101 (1 µm). Left panels: Real-Time PCR data comparing fold-difference in *CDKN1A and CLU* transcript in YUMAC and 501 mel cells after treatment with low-dose Aza and PXD101, alone and in combination, compared to non-treated cells. PXD101 (1 µM) was added for 24 hrs before harvesting the cells. Notice differences in scale of absolute hybridization intensities in YUMAC and 501 mel cells. However, 501 mel expressed about 370 fold less *CDKN1A* gene transcripts compared to YUMAC cells, apparently insufficient to lead to detectable p21^Cip1^ protein. The basal levels of *CLU* transcripts in YUMAC were about 50 fold higher relative to 501 mel melanoma cells, in agreement with low protein levels (data not shown). Panel C. Growth response to combination treatment with HDAC inhibitors Trichostatin A (TSA) and PXD101. The sensitive YUMAC and resistant 501 mel cells were incubated in triplicate wells without or with Aza (0.2 µM and 0.4 µM as indicated) followed by one-day recovery in regular medium, or medium supplemented with increasing concentrations of TSA (a), or PXD101 (b). Cell viability was assessed with the CellTiter-Glo® Luminescent Cell Viability Assay. Data are presented as percent of control, non-treated cells.

The global oligonucleotide gene expression data showed reactivation of TGFβ induced genes (*CLU* and *TGFBI*), which encode secreted proteins with potential to be markers for Aza-responsiveness. Clusterin levels in Aza treated cells were also assessed in the presence of MG132, because CLU, like p21^Cip1^, is sensitive to proteasomal degradation [36]. Western blot confirmed that protein levels corresponded, in general, to *CLU* transcripts, except for 501 mel cells which displayed reactivated gene transcripts with barely detectable protein, and YUSIT1 cells which expressed very little *CLU* mRNA but nevertheless exhibited high levels of Clusterin after inhibition with MG132 (**Figure 5A, B** **, relative mRNA levels confirmed by Real-Time RT-PCR,** **Figure 6D** **, and data not shown**). Nevertheless, the presence and absence of the protein correlated with the pattern of drug sensitivity and resistance, respectively, enhanced by blocking proteasomal degradation (**Figure 5B**). However, BS modified DNA sequencing showed that promoter methylation could not fully explain Clusterin basal and reactivated expression. *CLU* proximal promoter was methylated in a CpG rich island about 120 bp downstream of TSS, which underwent demethylation in response to Aza to the same extent in YUMAC, YUGEN8, WW165 and 501 mel cells (**Figure 5C**). Furthermore, the promoter was unmethylated in the non-expressing, untreated YURIF melanoma cells (**Figure 5C, YURIF**), results reminiscent of p21^Cip1^ activation.

### Synergistic reactivation with HDAC inhibitor

Aza reactivation of the unmethylated *CDKN1A* and *CLU* promoters suggested de-repression by methylation-independent mechanism. Because Aza can reverse histone-mediated silencing of unmethylated *CDKN1A* and other promoters [37], and the histone deacetylase (HDAC) inhibitor Trichostatin A (TSA) acts synergistically with Aza to reactivate hypomethylated promoters [38], we explored reactivation by TSA and the clinical HDAC inhibitor PXD101 [39], [40]. Indeed, TSA induced p21^Cip1^ and Clusterin in YUMAC cells in which the respective promoters are un- and hypo-methylated, respectively, in a dose dependent manner, even in the absence of MG132 (**Figure 6A, B, left panels**), but not p21^Cip1^ in 501 mel cells in which the promoter is fully methylated (data not shown). Likewise, p21^Cip1^ and Clusterin levels were increased in YUMAC and YURIF, but not in 501 mel cells, after overnight treatment with PXD101 (**Figure 6A, B, middle panels**). However, there was synergistic reactivation of p21^Cip1^ and Clusterin in 501 mel cells when PXD101 was combined with Aza (**Figure 6A, B middle panels**). These results, confirmed at the mRNA level by Real-Time PCR (**Figure 6A, B, right hand panels**), are consistent with the notion that the un- and hypomethylated promoters of these two genes are suppressed in melanoma cells by acetylated histone H3, and that Aza can release HDAC1 suppression and can act in synergy with HDAC inhibitors, as reported for AML and colorectal carcinoma cells [37].

The growth inhibitory effect of combination treatment of Aza with TSA and PDX101 were further explored and shown in **Figure 6Ca, b**. In YUMAC melanoma cells, CI-isobol analysis showed that TSA acted synergistically while PXD101 acted at most additive when combined with Aza (Figure S2) This suggests that PXD101 may induce cell arrest by other mechanisms, independent of gene re-expression [41].

### Reactivation of *TGFBI*

**Figure 7:**
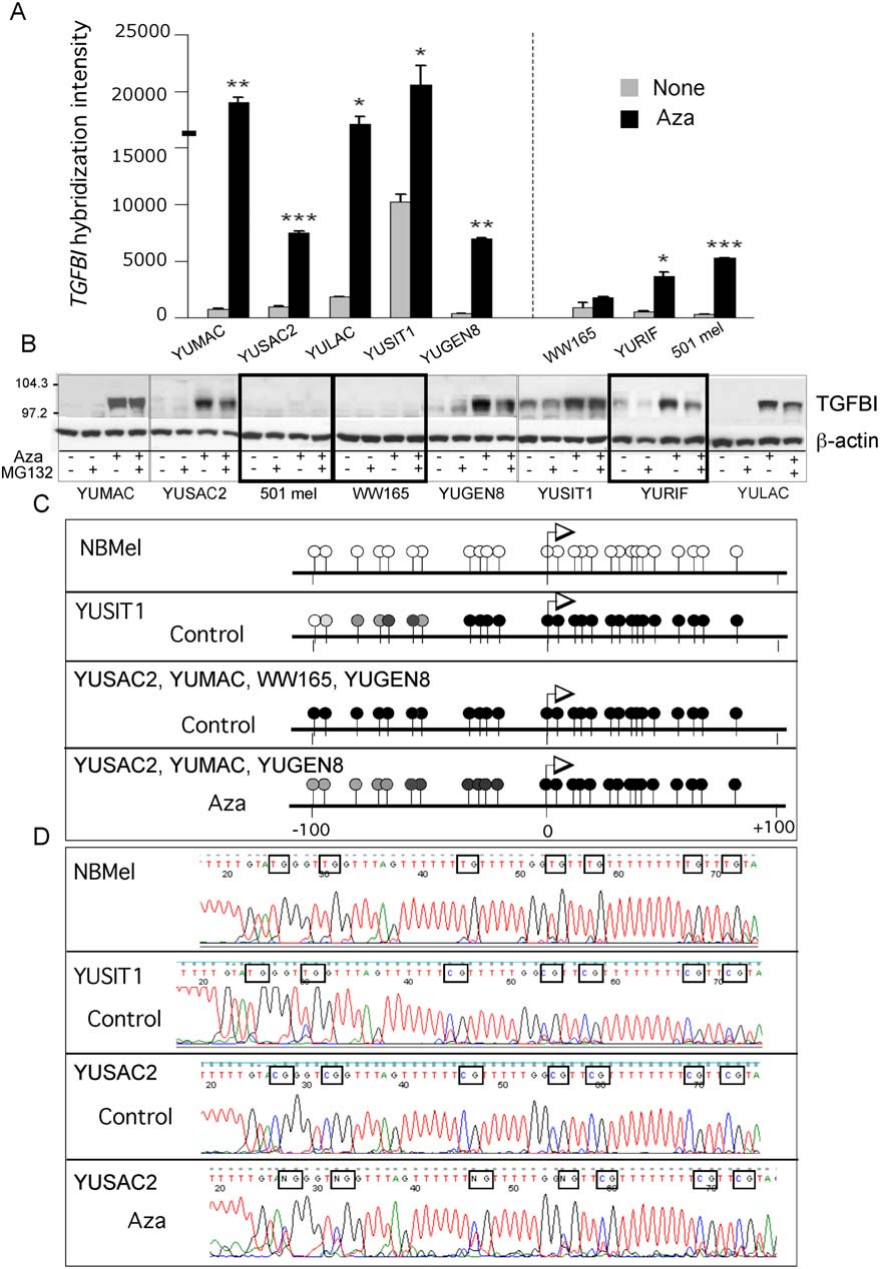
*TGFBI* reactivation by promoter demethylation. Panel A. *TGFBI* re-expression in response to Aza (0.2 µM) as assessed by the oligonucleotide array hybridization. The heavy line on the ordinate represent the levels of TGFBI- transcript levels in adult melanocytes. The data represent one sequence ID. All other details as in Figure 2 panel E. Panel B. Validation of TGFBI re-expression at the protein level by Western blots with anti-TGFBI antibodies employing b-actin as a control. The results are representative of two biological replicas. Panel C. BS sequencing results of *TGFBI* proximal promoter and first exon in normal human melanocytes (NBMel) and melanoma cells untreated (control), and Aza (0.2 µM) treated cells. Panel D. Chromatograms of the distal promoter about -50 to −100 bp downstream of TSS as shown in C. Boxed nucleotide pairs indicate position of intact (CG), partially BS modified (C/TG) and deaminated (TG) CG pairs. All other details as in Figure 4.

We were interested in reactivation of *TGFBI* (transforming growth factor, beta-induced, 68 kDa), because it is one of the novel genes that was not previously reported to be controlled by DNA methylation and it belongs to a set of ∼11 genes active in normal human melanocytes, silenced in melanoma cells, and reactivated by low-dose Aza (COL1A2, CTSK, GLB1L, IL11RA, MMP1, RND2, SERINC2, STC1, TNFRSF10D, FLJ22662) (**Figures 2B** **and** **7A**), and thus has the potential to serve as a marker for melanoma progression and responsiveness to Aza. The basal and Aza induced transcript levels of *TGFBI* were confirmed at the protein level (**Figure 7B**). However, unlike CLU, there was no complete separation between resistant and sensitive cells. Although *TGFBI* was not reactivated in two resistant cell strains (501 mel and WW165), it was induced in the third one, YURIF, to levels similar to those in sensitive cells (**Figure 7B**).

Sequencing of BS modified DNA revealed that *TGFBI* promoter was unmethylated and partially methylated in expressing normal human melanocytes and YUSIT1 melanoma cells, and completely methylated in non-expressing melanoma cells WW165, YUGEN8, YUMAC and YUSAC2 (**Figure 7C and D**). Furthermore, Aza caused demethylation in the three cell strains examined, YUSAC2, YUGEN8 and YUMAC, in which *TGFBI* was reactivated (**Figure 7C and D**). TGFBI promoter methylation was not restricted to metastatic cells or to cells in culture, because it was also methylated in primary melanoma cells freshly isolated from a 2.2 mm lesion (passage 1) and in five independent snap-frozen metastatic tumors (data not shown). These results suggest that *TGFBI* is indeed controlled by DNA methylation in melanoma cells and that promoter methylation may serve as a marker for malignant transformation.

**Figure 8:**
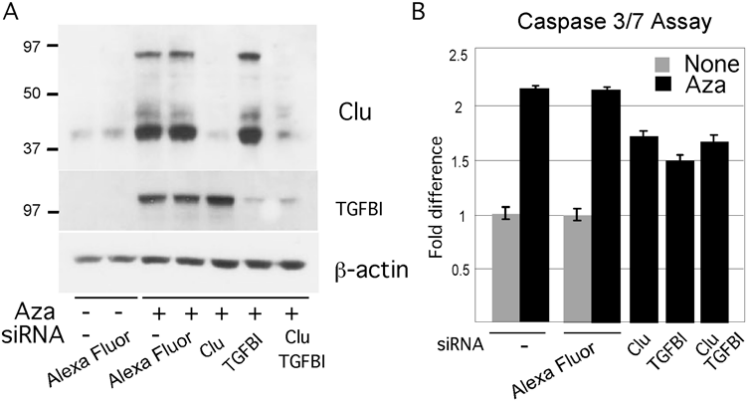
Validation of CLU and TGFBI apoptotic activity. Panel A. Reduction of Clu and TGBFI proteins by gene specific siRNA knockdown assessed by Western blotting. YUMAC melanoma cells were untreated or treated with Aza (0.2 µM) for 2 days followed by transient transfection with siRNA directed to Clu, TGFBI, or a mixture of the two, employing Alexa Fluor as a control, as indicated. Cells were harvested the following day and extracts subjected to successive Western blotting with the respective antibodies, and anti-β-actin as a control. Panel B. Parallel cultures were tested for apoptosis with the Caspase 3/7 assay. Values are given as percent of control, i.e., non-transfected cultures.

We assessed the contribution of the two TGFβ-pathway genes to the Aza apoptotic response the relatively sensitive YUMAC cell strain by short-term knockdown with gene-specific siRNA. *Clu* and *TGFBI* siRNA reduced the targeted protein to almost undetectable levels (**Figure 8A**). On the other hand, the apoptotic response of parallel cultures was reduced by 30% and 50% in Clu and TGFBI knockdown, respectively compared to Alexa fluor control transfectants, without any further increase in double knockdown cells (**Figure 8B**). These results indicated that Clu and TGFBI can account for some, but not all the apoptotic effect of Aza and that the two may act on the same pathway.

### Synergism between Aza and proteasomal inhibition

**Figure 9:**
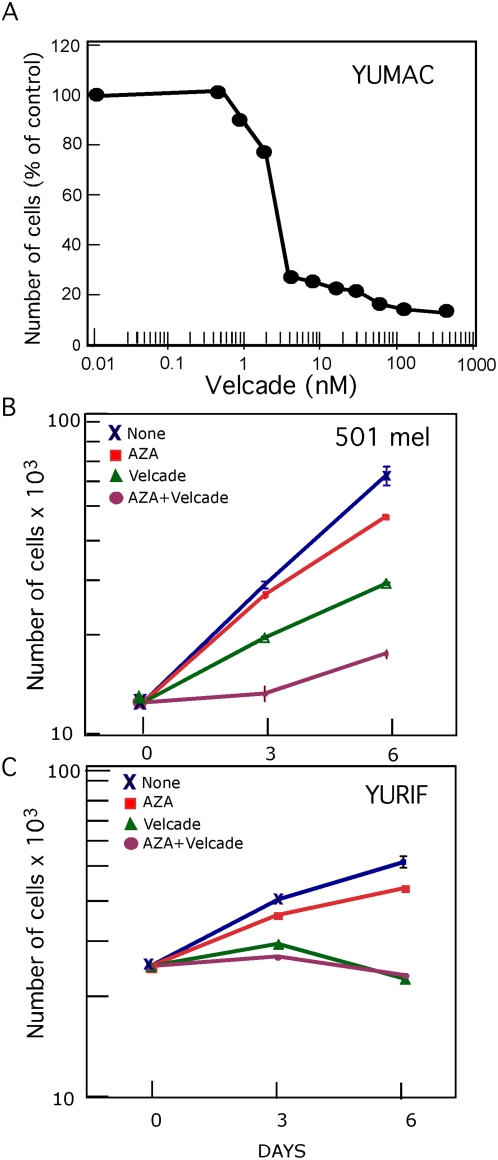
Bortezomib Augments the Aza growth-arrest response in a synergistic manner. Panel A. Dose-dependent effects of Bortezomib on cell proliferation. YUMAC melanoma cells were seeded in 24 well plates, incubated with increasing concentration of Bortezomib for 72 h, and cells from triplicate wells were harvested and counted with Coulter counter. Panels B and C. Growth responses of 501 mel and YURIF melanoma cells to 0.2 µM Aza (red), 2 nM Bortezomib (green) and combination (purple), compared to non-treated cells (black). Values are average of duplicate wells. Bars indicate double standard errors that ranged between 5–10% of total counts. Similar results were obtained with 1 nM Bortezomib in combination with Aza. The figures show one of two replicate experiments with similar results.

Guided by the observation that the two reactivated gene products Clusterin and p21^CIP^ were sensitive to proteasomal degradation, we tested if Bortezomib (Velcade), a reversible inhibitor of the 26S proteasome currently in clinical trials for cancer patients including melanoma [42], can enhance Aza growth inhibition, especially in resistant cells. Although the Cmax of Bortezomib at a standard dose and schedule (IV on days 1, 4, 8, 11 every 3 weeks) is high (80–500 ng/ml), it has a rapid distribution phase, and a terminal half-life of 9–10 hours. The clinical data suggest that Bortezomib levels drop to the low ng/ml range by several hours after dosing and remain at about 1 ng/ml (2.6 nM) for 24 hours. Bortezomib dose response showed that melanoma cells were highly sensitive to this inhibitor, with IC50 at the clinical relevant range of 2–3 nM as calculated by GraphPad Prism, and a steep curve at the of 1–4 nM range (**Figure 9A**). Low-doses of Bortezomib (2 nM) sensitized the resistant 501 mel melanoma cells to 0.2 µM Aza (**Figure 9B**). CI-isobol analysis showed that the drugs act synergistically (**Figure S2**). However, YURIF melanoma cells were even more sensitive to Bortezomib then YUMAC or 501 mel cells and there was no synergistic growth inhibition when the two drugs were combined (**Figure 9C**).

## Discussion

The results of our integrative examination of a panel of eight melanoma cell strains, three from short-term cultures, although in need of validation on a larger cohort, revealed underlying processes important for responsiveness to decitabine. The data implicated three major components in Aza responsiveness: a) activation of Wnt signaling; b) re-expression of p21^Cip1^ in a p53-independent manner and c) activation of two TGFβ pathway genes.

Comparing the gene expression profile of un-treated and treated melanoma cells implicated Wnt signaling based on high expression of the Wnt antagonist SFRP1 only in sensitive cells, which led us to further explore downstream members of this pathway and to identify activated β-catenin as a feature contributing to drug resistance. Although mutations in *CTNNB1* are rare in melanomas, activation might be through upstream modulators because a survey of large collection of melanoma tumors in tissue microarrays demonstrated that activated β-catenin in the nucleus is an independent predictor of poor survival [43]. The oncogenic potential of β-catenin was validated in a mouse model where stabilized β-catenin repressed p16^Ink4a^ expression and together with an activated NRas, lead to melanoma development with high penetrance and short latency [44]. We showed that the likely effect of activated β-catenin is upregulation of MITF, a potent melanocyte-specific transcription factor by itself considered an oncogene [45]. Interestingly only two of the three resistant cell strains, 501 mel and YURIF, harbored activated *CTNNB1* mutation. The third one, WW165 expressed constitutively high levels of endogenous MITF (in the absence of any gene amplification). Wnt/β-catenin pathway also interferes with responsiveness of CML to the tyrosine kinase inhibitor Imatinib [46], suggesting a common effect on other cancer cells as well. Therefore, various components of the activated Wnt/β pathway, in particular an activating mutation in β-catenin and high levels of MITF could be considered when selecting patients for this type of therapy, and devising combination therapy.

Protein analysis showed that p21^Cip1^ was upregulated in a p53 independent manner in two of the sensitive cell strains, but not in a resistant one. However, p21^Cip1^ was relatively stable and abundant in the other five melanoma cell strains, suggesting the emergence of resistance downstream of this cell cycle suppressor.

Whole genome expression analysis uncovered two reactivated TGFβ-responsive genes Clusterin and TGFBI that were more prominent in Aza-sensitive compared to resistant melanoma cells, and their activation enhanced apoptosis as observed by siRNA mediated gene knockdown. These two proteins can be used as markers, because they are secreted and have the potential to be released into the circulation. Furthermore, TGFBI promoter methylation might be useful as a marker for malignant transformation because it was unmethylated in normal melanocytes and hypo- or fully methylated in freshly isolated primary and metastatic melanoma cells, as well as melanoma tumors.

Our global gene expression analysis uncovered a total of 292 differentially expressed genes (mostly re-expression) across all melanoma strains after Aza treatment, with some products known to be associated with growth arrest. In addition to those described here, we validated the expression of UCHL1, PTPN6, TNFR1, SELENBP1, TNFR1, TNFRSF10D, S100A4, and several MAGE genes by semi-quantitative or real-time RT-PCR, or Western blots. Some of them, such as PTPN6 (protein tyrosine phosphatase, non-receptor type 6), that is expressed primarily in hematopoietic cells, were significantly induced at the protein level in the Aza sensitive YUMAC and YUSAC cells, but very little in the other cell types without any correlation to growth arrest or apoptotic response (**Supplementary Figure S3**). We surmise that other activated pathways, such as genes associated with acute inflammatory and immune responses or with activity on neighboring stroma cells, such as IGFBP5 [47], are likely to influence drug resistance *in vivo* and should be further explored.

We showed that gene reactivation by low-dose Aza in melanoma cells is through two known epigenetic activities of this drug, DNA promoter hypomethylation and histone modification. Other decitabine-responsive genes in our dataset, such as FN1, UCHL1, FUCA1, ICAM1, IL8, SERPINE2, TMEM45A and SFRP2 are also reactivated by HDAC inhibitors [48], [49], and might be modulated through histone modification by Aza as well. Aza can directly and indirectly modify histones as a function of DNMT status. DNMT1 interacts with HDAC1 [50] and elimination of DNMT1 displaces HDAC1 from target promoters [37]. In addition, Aza can affect histone methylation because DNMT1 binds also SUV39H1, a H3K9 methyltransferase, and EZH2 that catalyses the methylation of histone H3 at lysine 27 (H3K27), conferring a suppressive state [51]. Decitabine can also reduce the suppressive activity H3 K9 di-methylation by inducing changes in the transcription of enzymes responsible for this covalent modification [52]. Taken altogether, the observations reinforce the concept that impact on histone modification should be considered when dissecting the function of decitabine and devising combination therapy that is based on gene reactivation.

The protein validation data highlighted the importance of proteasomal degradation processes in responsiveness to Aza. At least two of the critical growth suppressor proteins, p21^Cip1^ and Clusterin, undergo proteasomal degradation. This observation led us to infer that a proteasomal inhibitor such as Bortezomib, currently in clinical trials, can synergize with low-dose Aza to alleviate resistance. This prediction was fulfilled in the case of 501 mel resistant cells. The synergistic response to this drug combination was unique because the Hsp90 inhibitor 17-AAG and the IGF1R inhibitor NVP-AEW541 (Novartis), employed at log-range of concentrations, did not show any synergistic growth arrest with Aza (data not shown).

Altogether, our results from this limited panel of melanoma cells suggest that treatment of melanoma patients could be improved by knowledge of the genetic and epigenetic background of individual tumors. In addition, they implicate that proteasomal and HDAC inhibitors might act in synergy with epigenetic modifiers for some patients.

## Materials and Methods

### Cells, drug treatments and proliferation assays

Normal human melanocytes were isolated from newborn foreskins and grown in OptiMEM (Invitrogen, Carlsbad, CA) with antibiotics, 5% fetal calf serum (regular medium) and growth supplements [53] and used during their first passage. Melanoma cells from primary and metastatic melanoma lesions (Table 1) were from tumor samples excised to improve patient quality of life. They were collected with participants' informed consent according to Health Insurance Portability and Accountability Act (HIPAA) regulations with Human Investigative Committee protocol. YUMAC, YULAC and YURIF melanoma cells were from short-term cultures (passage 2–15). The BRAF activating mutation was present in all the cell strains used in this study, two cell strains were null for PTEN, one expressed PTEN variant (Pro38Ser) but none harbored the N-Ras codon 61 mutation (**Table 1**). All primer sequences are available as supplementary data (**Table S2**).

Decitabine (5-Aza-2′-deoxy-cytidine, Sigma Chemical Co, St. Louis, MO, termed Aza) was dissolved in methanol as 10 mM stock solution, aliquoted and kept at −20°C. Dose response studies were performed with sparse melanoma cell cultures seeded in duplicate or triplicate wells (∼5,000 cells/cm^2^) in regular medium without or with increasing concentrations of Aza (0.1–1 µM) for 2 days, with fresh drug-containing medium on the second day. The cells were then released into drug-free medium, harvested at 2–3 days intervals and counted with the Coulter counter. The IC50 values for cell proliferation were calculated using the manual from the NIH Chemical Genomics Center (http://www.ncgc.nih.gov/guidance/section3.html). We defined the (inhibitive) response of a cell line to be the ratio of the population doubling time of the control (i.e., non-treated cells) to that of the treated cells. We assume that the Hill-Slope model of dose-response: y = 1/(1+(x/IC50)∧slope); y is the response corresponding to the dose x (Text S1).

Alternatively, viability was assessed with the CellTiter-Glo® Luminescent Cell Viability Assay (Promega Corporation, Madison, WI 53711) at the end of 3 days treatment, and the IC50 values were calculated by GraphPad Software, Inc., La Jolla, CA.

Trichostatin A (Sigma) was prepared as 3 mM stock solution diluted in ethanol. PXD101 (the Cancer Therapy Evaluation Program) was dissolved in DMSO as 10 mM stock solution and used at 1 µM. The effect of Bortezomib (from the oncology clinic pharmacy) on cell proliferation was assessed over 3 log concentrations (0.01–500 nM, in triplicate wells), for 72 hr, as described for Aza. Synergism between two drugs was estimated as described in the Supplementary method (Text S1).

### Apoptotic assays

Apoptosis was measured with the Caspase-Glo 3/7 assay kit from Promega following the manufacturer instructions. In addition, we used immunofluorescence with affinity-purified rabbit anti-caspase-3 active antibodies (AF835, R&D Systems) to assess the number of apoptotic cells after Aza treatment compared to controls. DAPI (4′,6-diamido-2-phenylindole dihydrochloride, Sigma Chemicals) was used to visualize nuclear DNA.

### Single cell DNA damage assay

The CometAssay Single Cell Gel Electrophoresis Assay kit (CometSlideTM, R&D Systems, Minneapolis, MN) was used to assess DNA damage in response to Aza following the manufacture's instructions. Briefly, melanoma cells were untreated or treated with Aza (0.2 µM, 0.5 µM and 1.0 µM) for 2 days followed by one day recovery as described above. As a positive control we used melanoma cells suspended in PBS and treated with 100 µM of hydrogen peroxide for 10 minutes on ice. Cells were harvested, re-suspended in PBS at 125,000 cells/ml and 50 µl portions were processed, subjected to electrophoreses and stained with SYBR Green I. Single fluorescing cells (100–120 cells from each treatment) were photographed, and analyzed with CASP software (http://casp.sourceforge.net). Quantitative and statistical data were generated by analysis of the results using the commercially available image analysis software packages that calculates tail length and tail moment termed CASP software (http://casp.sourceforge.net).

### RNA isolation and hybridization to DNA microarrays

Approximately 20–30 million cells (normal human melanocytes and melanoma cells/each) were used for mRNA extraction. The melanoma cells were treated with low-dose Aza (0.2 µM) for 2 days, followed by one day recovery, total RNA was extracted with the TRIzol reagent (Invitrogen Life Technologies, Inc., Invitrogen Corp., Carlsbad, CA), and Poly(A) mRNA was further isolated using the PolyATtract mRNA isolation system IV (Pro-mega, Madison, WI) following the manufacturer's instructions, and reversed transcribed to double stranded cDNA.

NimbleGen human whole genome expression microarrays (array 2005-04-20_Human_60mer_1in2) were used for hybridization. The same chip was hybridized with Cy3/Cy5 labeled polyA-selected cDNA from untreated and Aza treated melanoma cells. Each hybridization was repeated with dye swapping. The array hybridizations and data captures were performed by personnel at NimbleGen Systems Iceland LLC. Vínlandsleið 2–4, 113 Reykjavik, Iceland (currently Roche Applied Science, Basel, Switzerland).

### Bioinformatic analysis of global gene expression

#### Microarray design and data pre-processing

The NimbleGen oligonucleotide microarrays contain ∼380,000 probes with an average of 11 probes per sequence id. The entire set of sequence ids can be associated with ∼19,000 known genes. Normalization within arrays was performed with Loess-based methods to correct for biases due to labeling with different dyes on the two microarray channels. As such, M and A values were determined where M describes the amount of differential expression (M = log2(cy5/cy3)) and A associates M with the magnitude of overall expression (A = (log2cy5+log2cy3)/2). Normalization between arrays was performed via quantile-based methods to derive comparable A values (i.e., the average probe-signal). The steps of normalization within- and between-array were accomplished with tools provided in the *limma* Bioconductor library (8).

#### Selection of differentially expressed genes

A probe-level moderated *t*-statistic and the corresponding p-value were calculated via the limma library (9). In particular, an empirical Bayes method was employed to moderate the standard errors of the estimated log-fold changes, resulting in more stable inference and improved power (9). Multiple testing issues have been taken into account when determining the cutoff p-values. Next, we mapped probes to sequences by initially establishing a sequence's p-value distribution, and subsequently performing a *t*-test to determine whether this distribution was likely to have a mean of 1e-4 at an alpha level of 0.05. In effect, we are testing whether most of the probes had p-values below this threshold. Sequences that were significant, and whose probes were concordant in sign (i.e., no more than three discordant probes per sequence id) were retained. This pipeline was applied to identify differentially expressed genes after the Aza treatment (292 genes); and differentially expressed genes in untreated Aza resistant and sensitive cells (94 genes). WW165, 501 mel and YURIF were considered as the resistant, and the other five as sensitive strains for these analyses based on the IC50 values.

#### Functional grouping of differentially expressed genes

Differentially expressed sequences were evaluated for enrichment of GeneOntology (GO) terms, considering all the three ontologies: Molecular Functions, Cellular Component and Biological Processes (Harris et al., 2004). GO terms were assigned to each sequence id based on its Entrez gene id. A statistical test based on the hypergeometric distribution was used to determine the significance of the enrichment of each term. The final sets of GO terms were ranked based on their p-value and the most significant (p-value<1e-3) were selected.

#### Text mining

We performed text mining to better characterize genes that showed differential expression after Aza treatment. Specifically, we queried the literature for genes with known promoter hypermethylation in cancer, and for genes that have been shown to be regulated by treatment with epigenetic modifiers. We used a 2-step term mapping procedure called MarkIt [54] to properly flag a gene name with its appropriate Entrez Gene ID (http://www.ncbi.nlm.nih.gov/sites/entrez).

### Validation of gene expression

Protein levels were assessed by Western blots as described [55]. The membranes were probed with the following antibodies: anti-Clusterin (C-18, goat, sc-6419), anti-DNMT1 (K-18, goat, sc-10221), anti-p53 (pS20 sc-18079R rabbit), anti-p53 (pSer37, sc-28464-R), all from Santa Cruz Biotechnology CA; anti-MITF (clone D5) and anti-BCL2 (clone 124) mouse monoclonal antibodies from DAKO; anti-p53 (AF1355, goat) from R&D Systems; anti-p21^Cip1^ mAb (C24-4420) from BD Transduction Laboratories, Canada; anti-β-Actin mAb (A1978) from Sigma-Aldrich, St. Louis, MO 63103; anti-β-catenin (rabbit polyclonal from Dr. David Rimm, Pathology department, Yale University) [56]; and anti-TGFBI (rabbit polyclonal from Dr. Jan Johannes Enghild, diluted 1∶10,000).

Quantitative real-time RT-PCR was carried out in triplicate employing cDNA, using ABI 7500 Fast Real-Time PCR Systems and Power SYBRGreen (Applied Biosystems, Foster City, CA). The genes and primers used for probing are listed in **Table S2**. The expression of *ACTB* was used as a reference to normalize for input cDNA. The relative expression values were computed by the comparative Ct method.

### Downregulation of gene by siRNA

*CTNNB1* (β-catenin) was knock-downed with three different gene specific siRNA purchased from Qiagen, Valencia, CA as follows: CTCGGGATGTTCACAACCGAA (Hs_CTNNB1_5); CAGCGGCTTCTGCGCGACTTA (Hs_CTNNB1_8); CAGGATGATCCTAGCTATCGT (Hs_CTNNB1_9). Alexa Fluor 488 siRNA was used to monitor transfection efficiency as well as a control. An additional control was Allstars negative control siRNA from Qiagen (Cat number: 1027280). Melanoma cells were treated with Aza (0.2 µM) for 2 days and siRNAs were added at 10 nM employing the HiPerFect transfection reagent kit following the manufacturer instructions (Qiagen). The cells were harvested the following day and were assessed in parallel for protein expressions and apoptosis (triplicate wells). The extent of target gene knockdown (β-catenin), as well as downstream targets, MITF, Myc (β-catenin target genes), BCL2 (MITF target gene) [45], were assessed at protein levels by successive Western blotting with antibodies to β-catenin as a control.

Five different siRNA purchased from Qiagen were tested for Clusterin and TGFBI knockdown as revealed by Western blot analysis of the respected protein and one from each group was chosen for further experiments (CLU: ACAGACCTGCATGAAGTTCTA, and TGFBI: CGGGAAGGCGATCATCTCCAA) as described for *CTNNB1* knockdown.

### Analyses of proximal promoter methylation by bisulfite DNA sequencing

Genomic DNA (2 µg) was modified by sodium bisulfite (BS) and subjected to PCR amplification with primers that can bind to bisulfite treated DNA in non-CpG regions (**Table S1**) [53]. The amplified PCR products were gel-purified and the fragments were sequenced by Applied Biosystems 3730 capillary instruments at the W. M. Keck Foundation Biotechnology Resource Laboratory at Yale employing fluorescence-labeled dideoxynucleotides.

## Supporting Information

Figure S1 Growth responses of melanoma cells to increasing concentrations of Aza. Melanoma cells were untreated or treated with increasing concentrations of Aza for 2 days (underlined), released into regular growth medium and duplicate wells were counted at 2–3 days intervals. The Standard errors of most measurements were smaller then 10%, i.e., smaller than the symbols. Blue, none; Brown, 0.1 µM; Green, 0.2 µM; Red, 0.5 µM; and Black 1.0 µM. The results are representative of two biological replicas.
(0.14 MB TIF)

Figure S2 Isobologram of combination therapy of Decitabine (Aza) with Bortezomib, TSA and PDX in different melanoma cell strains. The colors correspond to particular drug combinations, and the individual points correspond to different drug dosages. If most points of a combination fall far below the additive effect line, then the combination is considered synergistic
(0.05 MB TIF)

Figure S3 PTPN6 activation in response to Aza. Panel A. PTPN6 expression in response to Aza (0.2 µM) as assessed by the oligonucleotide array hybridization. The data represent one sequence ID out of two with similar results. All other details as in Figure 2 panel E. Panel B. Validation of PTPN6 expression at the protein level by Western blotting with anti-PTPN6 mAb (anti-SHP-1 Ab-1 mAb, Lab Vision, Thermo Scientific, Fremont, CA), employing β-actin as a control. The results are representative of two biological replicas.
(0.59 MB TIF)

Text S1 (0.05 MB DOC)

Table S1 (0.04 MB DOC)

Table S2 (0.05 MB DOC)

## References

[1] John T, Black MA, Toro TT, Leader D, Gedye CA (2008). Predicting Clinical Outcome through Molecular Profiling in Stage III Melanoma.. Clin Cancer Res.

[2] Smalley KS, Contractor R, Nguyen TK, Xiao M, Edwards R (2008). Identification of a novel subgroup of melanomas with KIT/cyclin-dependent kinase-4 overexpression.. Cancer Res.

[3] Winnepenninckx V, Lazar V, Michiels S, Dessen P, Stas M (2006). Gene expression profiling of primary cutaneous melanoma and clinical outcome.. J Natl Cancer Inst.

[4] Viros A, Fridlyand J, Bauer J, Lasithiotakis K, Garbe C (2008). Improving melanoma classification by integrating genetic and morphologic features.. PLoS Med.

[5] Chin L, Garraway LA, Fisher DE (2006). Malignant melanoma: genetics and therapeutics in the genomic era.. Genes Dev.

[6] Curtin JA, Stark MS, Pinkel D, Hayward NK, Bastian BC (2006). PI3-kinase subunits are infrequent somatic targets in melanoma.. J Invest Dermatol.

[7] Baylin SB, Ohm JE (2006). Epigenetic gene silencing in cancer - a mechanism for early oncogenic pathway addiction?. Nat Rev Cancer.

[8] Feinberg AP (2008). Epigenetics at the epicenter of modern medicine.. Jama.

[9] Feinberg AP (2007). Phenotypic plasticity and the epigenetics of human disease.. Nature.

[10] Hoon DS, Spugnardi M, Kuo C, Huang SK, Morton DL (2004). Profiling epigenetic inactivation of tumor suppressor genes in tumors and plasma from cutaneous melanoma patients.. Oncogene.

[11] Baylin SB (2005). DNA methylation and gene silencing in cancer.. Nat Clin Pract Oncol.

[12] Esteller M (2008). Epigenetics in cancer.. N Engl J Med.

[13] Yoo CB, Jones PA (2006). Epigenetic therapy of cancer: past, present and future.. Nat Rev Drug Discov.

[14] Herranz M, Esteller M (2007). DNA methylation and histone modifications in patients with cancer: potential prognostic and therapeutic targets.. Methods Mol Biol.

[15] Jabbour E, Issa JP, Garcia-Manero G, Kantarjian H (2008). Evolution of decitabine development: accomplishments, ongoing investigations, and future strategies.. Cancer.

[16] Shang D, Liu Y, Matsui Y, Ito N, Nishiyama H (2008). Demethylating agent 5-aza-2′-deoxycytidine enhances susceptibility of bladder transitional cell carcinoma to Cisplatin.. Urology.

[17] Reu FJ, Bae SI, Cherkassky L, Leaman DW, Lindner D (2006). Overcoming resistance to interferon-induced apoptosis of renal carcinoma and melanoma cells by DNA demethylation.. J Clin Oncol.

[18] Bae SI, Cheriyath V, Jacobs BS, Reu FJ, Borden EC (2008). Reversal of methylation silencing of Apo2L/TRAIL receptor 1 (DR4) expression overcomes resistance of SK-MEL-3 and SK-MEL-28 melanoma cells to interferons (IFNs) or Apo2L/TRAIL.. Oncogene.

[19] Borden EC (2007). Augmentation of effects of interferon-stimulated genes by reversal of epigenetic silencing: potential application to melanoma.. Cytokine Growth Factor Rev.

[20] Zhu WG, Hileman T, Ke Y, Wang P, Lu S (2004). 5-aza-2′-deoxycytidine activates the p53/p21Waf1/Cip1 pathway to inhibit cell proliferation.. J Biol Chem.

[21] Wang H, Zhao Y, Li L, McNutt MA, Wu L (2008). An ATR signaling pathway and a phosphorylation-acetylation cascade are involved in activation of p53/p21WAF1/cip1 in response to 5-AZA-2′-deoxycytidine treatment.. J Biol Chem.

[22] Palii SS, Van Emburgh BO, Sankpal UT, Brown KD, Robertson KD (2008). The DNA methylation inhibitor 5-aza-2′-deoxycytidine (5-azadC) induces reversible genome-wide DNA damage that is distinctly influenced by DNA methyltransferases (DNMTs) 1 and 3B.. Mol Cell Biol.

[23] Chai G, Li L, Zhou W, Wu L, Zhao Y (2008). HDAC inhibitors act with 5-aza-2′-deoxycytidine to inhibit cell proliferation by suppressing removal of incorporated abases in lung cancer cells.. PLoS ONE.

[24] Gollob JA, Sciambi CJ, Peterson BL, Richmond T, Thoreson M (2006). Phase I trial of sequential low-dose 5-aza-2′-deoxycytidine plus high-dose intravenous bolus interleukin-2 in patients with melanoma or renal cell carcinoma.. Clin Cancer Res.

[25] Dahl C, Guldberg P (2007). The genome and epigenome of malignant melanoma.. Apmis.

[26] Liu S, Ren S, Howell P, Fodstad O, Riker AI (2008). Identification of novel epigenetically modified genes in human melanoma via promoter methylation gene profiling.. Pigment Cell Melanoma Res.

[27] Dahl E, Wiesmann F, Woenckhaus M, Stoehr R, Wild PJ (2007). Frequent loss of SFRP1 expression in multiple human solid tumours: association with aberrant promoter methylation in renal cell carcinoma.. Oncogene.

[28] Nojima M, Suzuki H, Toyota M, Watanabe Y, Maruyama R (2007). Frequent epigenetic inactivation of SFRP genes and constitutive activation of Wnt signaling in gastric cancer.. Oncogene.

[29] Schepsky A, Bruser K, Gunnarsson GJ, Goodall J, Hallsson JH (2006). The microphthalmia-associated transcription factor Mitf interacts with beta-catenin to determine target gene expression.. Mol Cell Biol.

[30] Rubinfeld B, Robbins P, El-Gamil M, Albert I, Porfiri E (1997). Stabilization of β-catenin by genetic defects in melanoma cell lines [see comments].. Science.

[31] Widlund HR, Horstmann MA, Price ER, Cui J, Lessnick SL (2002). Beta-catenin-induced melanoma growth requires the downstream target Microphthalmia-associated transcription factor.. J Cell Biol.

[32] Halaban R, Cheng E, Zhang Y, Moellmann G, Hanlon D (1997). Aberrant retention of tyrosinase in the endoplasmic reticulum mediates accelerated degradation of the enzyme and contributes to the dedifferentiated phenotype of amelanotic melanoma cells.. Proc Natl Acad Sci U S A.

[33] Tamm I, Wagner M, Schmelz K (2005). Decitabine activates specific caspases downstream of p73 in myeloid leukemia.. Ann Hematol.

[34] Schmelz K, Wagner M, Dorken B, Tamm I (2005). 5-Aza-2′-deoxycytidine induces p21WAF expression by demethylation of p73 leading to p53-independent apoptosis in myeloid leukemia.. Int J Cancer.

[35] Zheng QH, Ma LW, Zhu WG, Zhang ZY, Tong TJ (2006). p21Waf1/Cip1 plays a critical role in modulating senescence through changes of DNA methylation.. J Cell Biochem.

[36] Nizard P, Tetley S, Le Drean Y, Watrin T, Le Goff P (2007). Stress-induced retrotranslocation of clusterin/ApoJ into the cytosol.. Traffic.

[37] Scott SA, Dong WF, Ichinohasama R, Hirsch C, Sheridan D (2006). 5-Aza-2′-deoxycytidine (decitabine) can relieve p21WAF1 repression in human acute myeloid leukemia by a mechanism involving release of histone deacetylase 1 (HDAC1) without requiring p21WAF1 promoter demethylation.. Leuk Res.

[38] Cameron EE, Bachman KE, Myohanen S, Herman JG, Baylin SB (1999). Synergy of demethylation and histone deacetylase inhibition in the re-expression of genes silenced in cancer.. Nat Genet.

[39] Gimsing P, Hansen M, Knudsen LM, Knoblauch P, Christensen IJ (2008). A Phase I Clinical Trial of the Histone Deacetylase Inhibitor Belinostat (Pxd101) in Patients with Advanced Haematological Neoplasia.. Eur J Haematol.

[40] Steele NL, Plumb JA, Vidal L, Tjornelund J, Knoblauch P (2008). A phase 1 pharmacokinetic and pharmacodynamic study of the histone deacetylase inhibitor belinostat in patients with advanced solid tumors.. Clin Cancer Res.

[41] Glaser KB (2007). HDAC inhibitors: clinical update and mechanism-based potential.. Biochem Pharmacol.

[42] Orlowski RZ, Kuhn DJ (2008). Proteasome inhibitors in cancer therapy: lessons from the first decade.. Clin Cancer Res.

[43] Kielhorn E, Provost E, Olsen D, D'Aquila TG, Smith BL (2003). Tissue microarray-based analysis shows phospho-beta-catenin expression in malignant melanoma is associated with poor outcome.. Int J Cancer.

[44] Delmas V, Beermann F, Martinozzi S, Carreira S, Ackermann J (2007). beta-Catenin induces immortalization of melanocytes by suppressing p16INK4a expression and cooperates with N-Ras in melanoma development.. Genes Dev.

[45] Levy C, Khaled M, Fisher DE (2006). MITF: master regulator of melanocyte development and melanoma oncogene.. Trends Mol Med.

[46] Giles FJ, DeAngelo DJ, Baccarani M, Deininger M, Guilhot F (2008). Optimizing outcomes for patients with advanced disease in chronic myelogenous leukemia.. Semin Oncol.

[47] Chun T, Dong SM, Kim SH, Kang S, Seo SS (2008). Insulin-like growth factor binding protein-5 (IGFBP-5) acts as a tumor suppressor by inhibiting angiogenesis.. Carcinogenesis.

[48] Hellebrekers DM, Melotte V, Vire E, Langenkamp E, Molema G (2007). Identification of epigenetically silenced genes in tumor endothelial cells.. Cancer Res.

[49] Sova P, Feng Q, Geiss G, Wood T, Strauss R (2006). Discovery of novel methylation biomarkers in cervical carcinoma by global demethylation and microarray analysis.. Cancer Epidemiol Biomarkers Prev.

[50] Fuks F, Burgers WA, Brehm A, Hughes-Davies L, Kouzarides T (2000). DNA methyltransferase Dnmt1 associates with histone deacetylase activity.. Nat Genet.

[51] Vire E, Brenner C, Deplus R, Blanchon L, Fraga M (2006). The Polycomb group protein EZH2 directly controls DNA methylation.. Nature.

[52] Wozniak RJ, Klimecki WT, Lau SS, Feinstein Y, Futscher BW (2007). 5-Aza-2′-deoxycytidine-mediated reductions in G9A histone methyltransferase and histone H3 K9 di-methylation levels are linked to tumor suppressor gene reactivation.. Oncogene.

[53] Cheng E, Trombetta SE, Kovacs D, Beech RD, Ariyan S (2006). Rab33A: characterization, expression, and suppression by epigenetic modification.. J Invest Dermatol.

[54] Luong T, Tran N, Krauthammer M (2007). Context-Aware Mapping of Gene Names using Trigrams..

[55] Hoek K, Rimm DL, Williams KR, Zhao H, Ariyan S (2004). Expression profiling reveals novel pathways in the transformation of melanocytes to melanomas.. Cancer Res.

[56] Rimm DL, Caca K, Hu G, Harrison FB, Fearon ER (1999). Frequent nuclear/cytoplasmic localization of beta-catenin without exon 3 mutations in malignant melanoma.. Am J Pathol.

