# Supplementary figures and images for: Integrative Analysis of Epigenetic Modulation in Melanoma Cell Response to Decitabine: Clinical Implications
Source: PLoS One. 2009 Feb 23;4(2):e4563. doi: 10.1371/journal.pone.0004563 (PMC2642998; doi:10.1371/journal.pone.0004563)

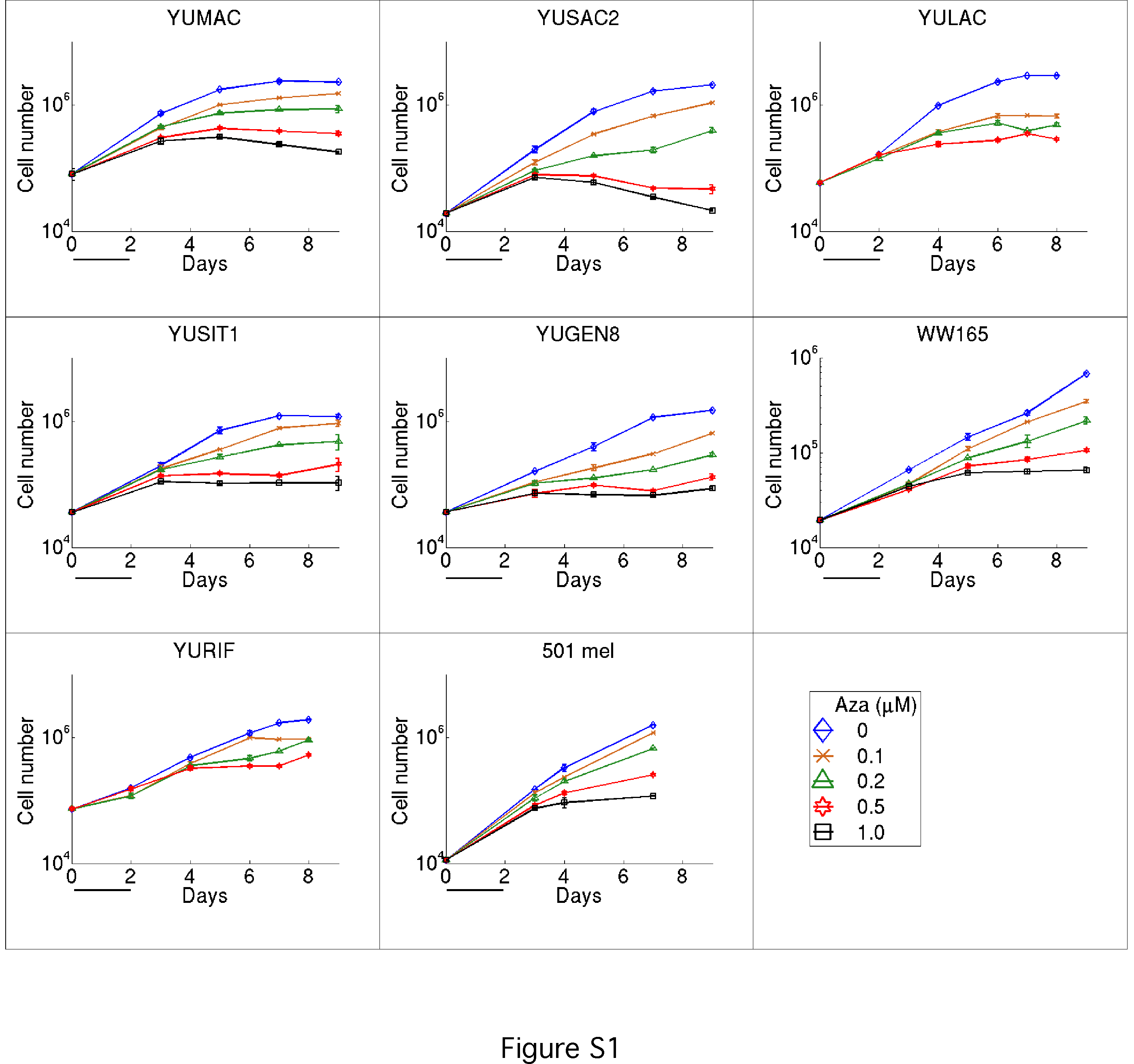

Supplement: Figure S1 — Growth responses of melanoma cells to increasing concentrations of Aza. Melanoma cells were untreated or treated with increasing concentrations of Aza for 2 days (underlined), released into regular growth medium and duplicate wells were counted at 2–3 days intervals. The Standard errors of most measurements were smaller then 10%, i.e., smaller than the symbols. Blue, none; Brown, 0.1 µM; Green, 0.2 µM; Red, 0.5 µM; and Black 1.0 µM. The results are representative of two biological replicas. (0.14 MB TIF) [file pone.0004563.s001.tif]

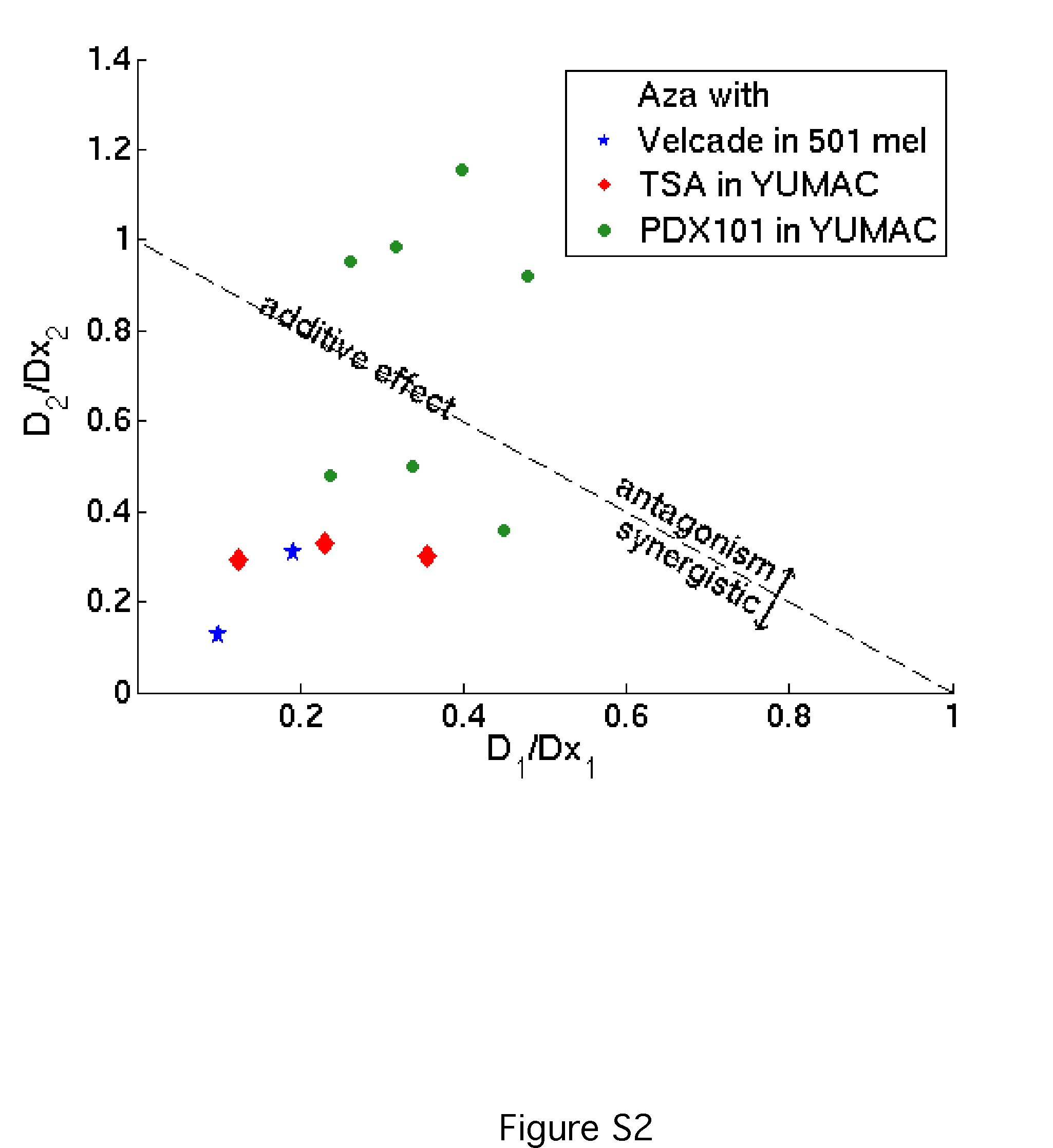

Supplement: Figure S2 — Isobologram of combination therapy of Decitabine (Aza) with Bortezomib, TSA and PDX in different melanoma cell strains. The colors correspond to particular drug combinations, and the individual points correspond to different drug dosages. If most points of a combination fall far below the additive effect line, then the combination is considered synergistic (0.05 MB TIF) [file pone.0004563.s002.tif]

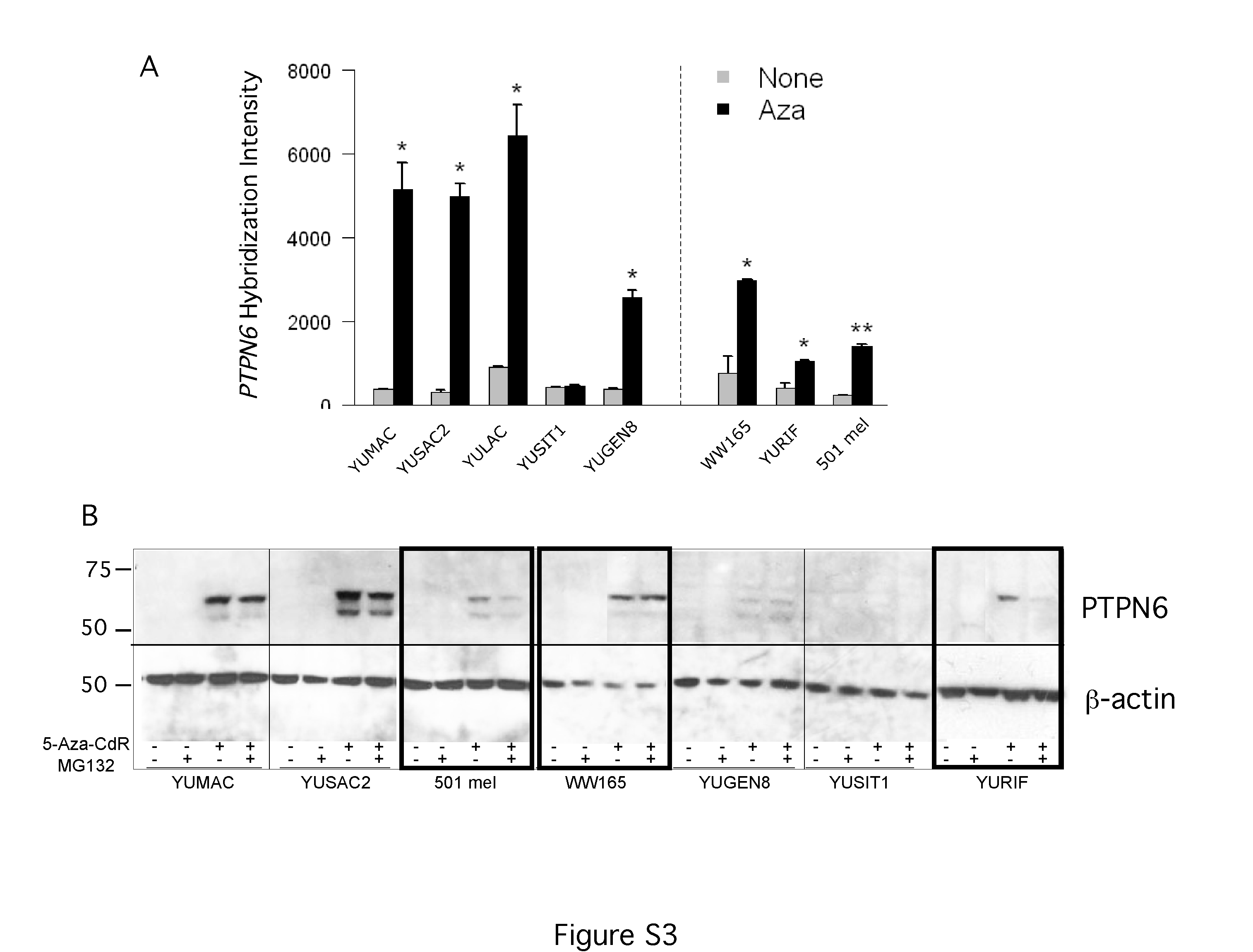

Supplement: Figure S3 — PTPN6 activation in response to Aza. Panel A. PTPN6 expression in response to Aza (0.2 µM) as assessed by the oligonucleotide array hybridization. The data represent one sequence ID out of two with similar results. All other details as in Figure 2 panel E. Panel B. Validation of PTPN6 expression at the protein level by Western blotting with anti-PTPN6 mAb (anti-SHP-1 Ab-1 mAb, Lab Vision, Thermo Scientific, Fremont, CA), employing β-actin as a control. The results are representative of two biological replicas. (0.59 MB TIF) [file pone.0004563.s003.tif]
