# Supplementary material for: Integrative Analysis of Epigenetic Modulation in Melanoma Cell Response to Decitabine: Clinical Implications
Source: PLoS One. 2009 Feb 23;4(2):e4563. doi: 10.1371/journal.pone.0004563 (PMC2642998; doi:10.1371/journal.pone.0004563)
Supplement: Text S1 — (0.05 MB DOC) [file pone.0004563.s004.doc]

**Statistical methods**

We used methods of Chou-Talalay [1] to compute IC50s and determine synergism. IC50s are computed from the median effect equation. Synergism analysis is carried out using the CI-isobol method.

Median-effect equation

The median-effect equation models of the effect of an inhibitor (such as a drug) as

*F_a_/F_u_ = (D/D50)^m*

where *D* is the dose, *F_a_* and *F_u_* is the fraction of the system affected and unaffected by the dose *D* (*F_a_ + F_u_ = 1); D50* is the dose producing the median effect (e.g. IC50, ED50, LD50). The constant *m* determines the shape of the dose-effect curve.

The median-effect equation in logarithm has the form

*log(F_a_/F_u_) = m log(D) - m log(D50)*

which essentially represents a linear relationship between *log(F_a_/F_u_)* and *log(D)*. Thus, given a data of doses and their corresponding effects (i.e. dose-effect data), we can carry out a linear regression calculation to estimate the parameters *m* and *D50*.

We apply the median effect equation to calculate the IC50s of the 8 cell lines as follows. The effects of decitabine on the cell lines are measured through changes in their population doubling time (PDT). We define the fraction *F_u_* to be the ratio of the PDT of a treated cell line to the PDT of a control, which is the cell line without the treatment. That is:

*F_u_ = PDT(treated cell line)/PDT(non-treated cell line)*

Then the IC50 of a cell line is the constant *D50* in the median effect equation, which can be estimated by the linear regression described above.

CI-isobol method

The CI-isobol method provides a quantitative assessment of synergism between drugs. A combination index (CI) is estimated from dose-effect data of single and combined drug treatments. A value of CI less than 1 indicates synergism; CI = 1 indicates additive effect; and CI > 1 indicates antagonism. Drug interaction (synergism or antagonism) is more pronounced the farther a CI value is from 1.

Formally, the combination index (CI) of a combined drug treatment is defined as

*CI = D_1_/ Dx_1_ + D_2_/ Dx_2_*

Here *D_1_* and *D_2_* are the doses of drug *1* and drug *2, respectively,* in the combination; *Dx_1_* and *Dx_2_* each is the dose of a treatment with only drug *1* and drug *2* that would give the same effect as that of the combination, respectively. The doses *Dx_1_* and *Dx_2_* need to be estimated from the dose-effect data of single drug treatments. Essentially, a median effect equation is fitted to the data of each drug. From the median effect equation of a drug, we can estimate the dose (i.e. *D)* necessary to produce an effect (i.e. *F_a_, F_u_*). The results can be summarized in a normalized isobologram. A point in the isobologram represents the effect of a drug treatment. The further a point lies from the additive line, the bigger the different between 1 and its CI, thus the stronger the (synergistic or antagonistic) effect is.

**Reference**

1. Chou TC, Talalay P (1984) Quantitative analysis of dose-effect relationships: the combined effects of multiple drugs or enzyme inhibitors. Adv Enzyme Regul 22: 27-55.
