# Supplementary material for: Integrative Analysis of Epigenetic Modulation in Melanoma Cell Response to Decitabine: Clinical Implications
Source: PLoS One. 2009 Feb 23;4(2):e4563. doi: 10.1371/journal.pone.0004563 (PMC2642998; doi:10.1371/journal.pone.0004563)
Supplement: Table S1 — (0.04 MB DOC) [file pone.0004563.s005.doc]

Table S1: Melanoma cell proliferation in response to increasing concentrations of Aza

| Melanoma | Aza (µM) | | | | |
| --- | --- | --- | --- | --- | --- |
|  | 0 | 0.1 | 0.2 | 0.5 | 1.0 |
| YUMAC | 1.1 | 6.8 | 17.7 | Arrest | Arrest |
| YUSAC2 | 0.9 | 1.9 | 3.0 | Arrest | Arrest |
| YULAC | 1.1 | 1.9 | 9.3 | Arrest | Arrest |
| YUSIT1 | 1.2 | 1.9 | 3.9 | Arrest | Arrest |
| YUGEN8 | 1.4 | 2.2 | 4.0 | Arrest | Arrest |
| WW165 | 1.8 | 2.4 | 3.0 | 7.1 | Arrest |
| YURIF | 1.5 | 1.9 | 2.2 | 6.8 | Arrest |
| 501mel | 1.2 | 1.3 | 1.6 | 2.6 | 6.6 |

Melanoma cells were untreated or treated with increasing concentrations of Aza for 2 days, released into fresh growth medium and counted at 1-3 days intervals. The numbers represent Population Doubling Time (PDT) in days. PDTs were calculated using a model of exponential growth. We have used the PDT from the first several days of growth in untreated cells, and over the last days of growth in drug-treated cells. Growth response curves of all cell strains are presented in **Figure S1.**
