# Supplementary material for: Integrative Analysis of Epigenetic Modulation in Melanoma Cell Response to Decitabine: Clinical Implications
Source: PLoS One. 2009 Feb 23;4(2):e4563. doi: 10.1371/journal.pone.0004563 (PMC2642998; doi:10.1371/journal.pone.0004563)
Supplement: Table S2 — (0.05 MB DOC) [file pone.0004563.s006.doc]

Table S2: Primers used for PCR amplifications

| Gene | Purpose | Primer sequence | Fragment size (bp) |
| --- | --- | --- | --- |
| *CDKNA1* | Promoter methylation | F: 5'-AGGAGGGAAGTGTTTTTTTGTAGTA-3'  R: 5'-ACAACTACTCACACCTCAACTAAC-3' | 235 |
| *TGFBI* | Promoter methylation | F: 5’-GGGTGGGTGTTTAGGGTAGTTA-3’  R: 5’-AACCTACTATACTACAACACCAAC-3' | 335 |
| *CLU* | Promoter methylation | F: 5'-GTTAGTAGGGTTAGGGAATTGTGAG-3'  R: 5'-ACACACCCCCTTTAAAACTAACTAC-3' | 312 |
| *TP53* | Mutation | F1: 5'-TGACACGCTTCCCTGGATTG-3’  R1: 5'-GCACAAACACGCACCTCAAAG-3' | 876 |
|  |  | F2: 5'-TGGTAATCTACTGGGACGGAACAG-3'  R2: 5'-GCTTCTGACGCACACCTATTGC-3' | 514 |
| *BRAF* | Mutation (Exon 15) | F: 5'-CTACTGTTTTCCTTTACTTACTACACCTCAGA-3'  R: 5'-AACTCAGCAGCATCTCAGGGC-3' | 430 |
| *NRAS* | Mutation (Exon 3) | F: 5' CACACCCCCAGGATTCTTAC-3'  R: 5'-TGGCAAATACACAGAGGAAGC-3' | 150 |
| *PTEN* | Mutation, cDNA | F1: 5’- TGCCATCTCTCTCCTCCTTTTTC-3  R1: 5’-CCTCTGGTCCTGGTATGAAGAAT-3 | 897 |
|  |  | F2: 5'- GAGTAACTATTCCCAGTCAGAGGCG-3'  R2: 5'-CAAGTGTCAAAACCCTGTGGATG-3' | 896 |
| *CTNNB1* | Mutation | F: 5'-TGGAACCAGACAGAAAAGCGG-3'  R: 5'-TGAGTGAAGGACTGAGAAAATCCC-3’ | 187 |
| *CLU* | qPCR | F: :5'-TGTCCCGGCTGGCAAA-3'  R: 5'-CGTGGTGACCCGCAGATAGT-3’ | 59 |
| *CDKN1A* | qPCR | F: 5'-GCGGCAGACCAGCATGA-3’  R: 5'-ATTAGGGCTTCCTCTTGGAGAAG-3’ | 71 |
| *TGFBI* | qPCR | F: 5'-TCCACAGCCATTGACCTTTTC-3’  R: 5’-TCAACCGCTCACTTCCAGAGA-3’ | 67 |

F and R: Forward and Reverse primers
